# Evaluating the transcriptional fidelity of cancer models

**DOI:** 10.1186/s13073-021-00888-w

**Published:** 2021-04-29

**Authors:** Da Peng, Rachel Gleyzer, Wen-Hsin Tai, Pavithra Kumar, Qin Bian, Bradley Isaacs, Edroaldo Lummertz da Rocha, Stephanie Cai, Kathleen DiNapoli, Franklin W. Huang, Patrick Cahan

**Affiliations:** 1 Department of Biomedical Engineering, Johns Hopkins University School of Medicine, Baltimore, MD 21205 USA; 2 Institute for Cell Engineering, Johns Hopkins University School of Medicine, Baltimore, MD 21205 USA; 3 Department of Microbiology, Immunology and Parasitology, Federal University of Santa Catarina, Florianópolis, SC Brazil; 4 Department of Cell Biology, Johns Hopkins University School of Medicine, Baltimore, MD 21205 USA; 5 Department of Electrical and Computer Engineering, Johns Hopkins University, Baltimore, MD 21218 USA; 6 Division of Hematology/Oncology, Department of Medicine; Helen Diller Family Cancer Center; Bakar Computational Health Sciences Institute; Institute for Human Genetics, University of California, San Francisco, San Francisco, CA USA; 7 Department of Molecular Biology and Genetics, Johns Hopkins University School of Medicine, Baltimore, MD 21205 USA

**Keywords:** Cancer models, Machine learning, Cancer cell lines, PDX, GEMM, Tumoroid, Tumor classification

## Abstract

**Background:**

Cancer researchers use cell lines, patient-derived xenografts, engineered mice, and tumoroids as models to investigate tumor biology and to identify therapies. The generalizability and power of a model derive from the fidelity with which it represents the tumor type under investigation; however, the extent to which this is true is often unclear. The preponderance of models and the ability to readily generate new ones has created a demand for tools that can measure the extent and ways in which cancer models resemble or diverge from native tumors.

**Methods:**

We developed a machine learning-based computational tool, CancerCellNet, that measures the similarity of cancer models to 22 naturally occurring tumor types and 36 subtypes, in a platform and species agnostic manner. We applied this tool to 657 cancer cell lines, 415 patient-derived xenografts, 26 distinct genetically engineered mouse models, and 131 tumoroids. We validated CancerCellNet by application to independent data, and we tested several predictions with immunofluorescence.

**Results:**

We have documented the cancer models with the greatest transcriptional fidelity to natural tumors, we have identified cancers underserved by adequate models, and we have found models with annotations that do not match their classification. By comparing models across modalities, we report that, on average, genetically engineered mice and tumoroids have higher transcriptional fidelity than patient-derived xenografts and cell lines in four out of five tumor types. However, several patient-derived xenografts and tumoroids have classification scores that are on par with native tumors, highlighting both their potential as faithful model classes and their heterogeneity.

**Conclusions:**

CancerCellNet enables the rapid assessment of transcriptional fidelity of tumor models. We have made CancerCellNet available as a freely downloadable R package (https://github.com/pcahan1/cancerCellNet) and as a web application (http://www.cahanlab.org/resources/cancerCellNet_web) that can be applied to new cancer models that allows for direct comparison to the cancer models evaluated here.

**Supplementary Information:**

The online version contains supplementary material available at 10.1186/s13073-021-00888-w.

## Background

Models are widely used to investigate cancer biology and to identify potential therapeutics. Popular modeling modalities are cancer cell lines (CCLs) [1], genetically engineered mouse models (GEMMs) [2], patient-derived xenografts (PDXs) [3], and tumoroids [4]. These classes of models differ in the types of questions that they are designed to address. CCLs are often used to address cell-intrinsic mechanistic questions [5], GEMMs to chart the progression of molecularly defined-disease [6], and PDXs to explore patient-specific response to therapy in a physiologically relevant context [7]. More recently, tumoroids have emerged as relatively inexpensive, physiological, in vitro 3D models of tumor epithelium with applications ranging from measuring drug responsiveness to exploring tumor dependence on cancer stem cells [4]. Models also differ in the extent to which they represent specific aspects of a cancer type. Even with this intra- and inter-class model variation, all models should represent the tumor type or subtype under investigation, and not another type of tumor, and not a non-cancerous tissue. Therefore, cancer models should be selected not only based on the specific biological question but also based on the similarity of the model to the cancer type under investigation [8, 9].

Various methods have been proposed to determine the similarity of cancer models to their intended subjects. Domcke et al. devised a ‘suitability score’ as a metric of the molecular similarity of CCLs to high-grade serous ovarian carcinoma based on a heuristic weighting of copy number alterations, mutation status of several genes that distinguish ovarian cancer subtypes, and hypermutation status [10]. Other studies have taken analogous approaches by either focusing on transcriptomic or ensemble molecular profiles (e.g. transcriptomic and copy number alterations) to quantify the similarity of cell lines to tumors [11–13]. These studies were tumor-type specific, focusing on CCLs that model, for example, hepatocellular carcinoma or breast cancer. Notably, Yu et al. compared the transcriptomes of CCLs to The Cancer Genome Atlas (TCGA) by correlation analysis, resulting in a panel of CCLs recommended as most representative of 22 tumor types [14]. Most recently, Najgebauer et al. [15] and Salvadores et al. [16] have developed methods to assess CCLs using molecular traits such as copy number alterations (CNA), somatic mutations, DNA methylation, and transcriptomics. While all of these studies have provided valuable information, they leave two major challenges unmet. The first challenge is to determine the fidelity of GEMMs, PDXs, and tumoroids, and whether there are stark differences between these classes of models and CCLs. The other major unmet challenge is to enable the rapid assessment of new, emerging cancer models. This challenge is especially relevant now as technical barriers to generating models have been substantially lowered [17, 18], and because new models such as PDXs and tumoroids can be derived on patient-specific basis and therefore should be considered distinct entities requiring individual validation [4, 19].

To address these challenges, we developed CancerCellNet (CCN), a computational tool that uses transcriptomic data to quantitatively assess the similarity between cancer models and 22 naturally occurring tumor types and 36 subtypes in a platform- and species-agnostic manner. Here, we describe CCN’s performance, and the results of applying it to assess 657 CCLs, 415 PDXs, 26 GEMMs, and 131 tumoroids. This has allowed us to identify the most faithful models currently available, to document cancers underserved by adequate models, and to find models with an inaccurate tumor type annotation. Moreover, because CCN is open-source and easy to use, it can be readily applied to newly generated cancer models as a means to assess their fidelity.

## Methods

### Online methods

#### Training general CancerCellNet classifier

We downloaded 8991 patient tumor RNA-seq expression count matrix, generated by TCGA research Network [20]: https://www.cancer.gov/tcga and their corresponding sample table across 22 different tumor types using TCGAWorkflowData, TCGAbiolinks [21], and SummarizedExperiment [22] packages. We used all the patient tumor samples for training the general CCN classifier. We limited training and analysis of RNA-seq data to the 13,142 genes in common between the TCGA dataset and all the query samples (CCLs, PDXs, GEMMs, and tumoroids). To train the top pair Random Forest classifier, we used a method similar to our previous method [23]. CCN first normalized the training counts matrix by down-sampling the counts to 500,000 counts per sample. To significantly reduce the execution time and memory of generating gene pairs for all possible genes, CCN then selected *n* upregulated genes, *n* downregulated genes, and *n* least differentially expressed genes (CCN training parameter nTopGenes = *n*) for each of the 22 cancer categories using template matching [24] as the genes to generate top-scoring gene pairs. In short, for each tumor type, CCN defined a template vector that labeled the training tumor samples in cancer type of interest as 1 and all other tumor samples as 0. CCN then calculated the Pearson correlation coefficient between template vector and gene expressions for all genes. The genes with a strong match to the template as either upregulated or downregulated had a large absolute Pearson correlation coefficient. CCN chose the upregulated, downregulated, and least differentially expressed genes based on the magnitude of the Pearson correlation coefficient.

After CCN selected the genes for each cancer type, CCN generated gene pairs among those genes. Gene pair transformation was a method inspired by the top-scoring pair classifier [25] to allow compatibility of the classifier with query expression profiles that were collected through different platforms (e.g. microarray query data applied to RNA-seq training data). In brief, the gene pair transformation compares 2 genes within an expression sample and encodes the “gene1_gene2” gene-pair as 1 if the first gene has higher expression than the second gene. Otherwise, gene pair transformation would encode the gene-pair as 0. Using all the gene pair combinations generated through the gene sets per cancer type, CCN then selected top *m* discriminative gene pairs (CCN training parameter nTopGenePairs = *m*) for each category using template matching (with large absolute Pearson correlation coefficient) described above. To prevent any single gene from dominating the gene pair list, we allowed each gene to appear at a maximum of three times among the gene pairs selected as features per cancer type.

After the top discriminative gene pairs were selected for each cancer category, CCN grouped all the gene pairs together and gene pair transformed the training samples into a binary matrix with all the discriminative gene pairs as row names and all the training samples as column names. Using the binary gene pair matrix, CCN randomly shuffled the binary values across rows then across columns to generate random profiles that should not resemble training data from any of the cancer categories. CCN then sampled 70 random profiles, annotated them as “Unknown,” and used them as training data for the “Unknown” category. Using gene pair binary training matrix, CCN constructed a multi-class Random Forest classifier of 2000 trees and used a stratified sampling of 60 sample sizes to ensure a balance of training data in constructing the decision trees.

To identify the best set of genes and gene-pair parameters (*n* and *m*), we used a grid-search cross-validation [26] strategy with 5 cross-validations at each parameter set. The specific parameters for the final CCN classifier using the function “broadClass_train” in the package cancerCellNet are in Additional file 1: Table S1. The gene pairs are in Additional file 2: Table S2.

### Validating general CancerCellNet classifier

Two thirds of patient tumor data from each cancer type were randomly sampled as training data to construct a CCN classifier. Based on the training data, CCN selected the classification genes and gene pairs and trained a classifier. After the classifier was built, 35 held-out samples from each cancer category were sampled and 40 “Unknown” profiles were generated for validation. The process of randomly sampling training set from 2/3 of all patient tumor data, selecting features based on the training set, training classifier, and validating was repeated 50 times to have a more comprehensive assessment of the classifier trained with the optimal parameter set. To test the performance of final CCN on independent testing data, we applied it to 725 profiles from ICGC spanning 6 projects that do not overlap with TCGA (BRCA-KR, LIRI-JP, OV-AU, PACA-AU, PACA-CA, PRAD-FR).

### Selecting decision thresholds

Our strategy for selecting a decision threshold was to find the value that maximizes the average Macro F1 measure [27] for each of the 50 cross-validations that were performed with the optimal parameter set, testing thresholds between 0 and 1 with a 0.01 increment. The F1 measure is defined as:

$$ \mathrm{Macro}\ \mathrm{F}1=\frac{2\times \mathrm{precision}\times \mathrm{recall}}{\mathrm{precision}+\mathrm{recall}} $$

We selected the most commonly occurring threshold above 0.2 that maximized the average Macro F1 measure across the 50 cross-validations as the decision threshold for the final classifier (threshold = 0.25). The same approach was applied for the subtype classifiers. The thresholds and the corresponding average precision, recall, and F1 measures are recorded in (Additional file 3: Table S3).

### Classifying query data into general cancer categories

We downloaded the RNA-seq cancer cell line expression profiles (CCLE_RNAseq_genes_counts_20180929.gct released on 02-Jan-2019) and sample table (Cell_lines_annotations_20181226.txt released on 11-Feb-2019) from (https://portals.broadinstitute.org/ccle/data), and microarray cancer cell line expression profiles and sample table from Barretina et al. [28]. We extracted two WT control NCCIT RNA-seq expression profiles from Grow et al. [29]. We received PDX expression estimates and sample annotations from the authors of Gao et al. [19]. We gathered GEMM expression profiles from nine different studies [30–38]. We downloaded tumoroid expression profiles from the NCI patient-derived models repository (PDMR) [39] and from three individual studies [40–42]. To use CCN classifier on GEMM data, the mouse genes from GEMM expression profiles were converted into their human orthologs. The query samples were classified using the final CCN classifier. Each query classification profile was labeled as one of the four classification categories: “correct,” “mixed,” “none,” and “other” based on classification profiles. If a sample has a CCN score higher than the decision threshold in the labeled cancer category, we assigned that as “correct.” If a sample has CCN score higher than the decision threshold in labeled cancer category and in other cancer categories, we assigned that as “mixed.” If a sample has no CCN score higher than the decision threshold in any cancer category or has the highest CCN score in “Unknown” category, then we assigned it as “none.” If a sample has CCN score higher than the decision threshold in a cancer category or categories not including the labeled cancer category, we assigned it as “other.” We analyzed and visualized the results using R and R packages pheatmap [43] and ggplot2 [44].

### Cross-species assessment

To assess the performance of cross-species classification, we downloaded 1003 labeled human tissue/cell type and 1993 labeled mouse tissue/cell type RNA-seq expression profiles from Radley et al. [45] (https://github.com/pcahan1/CellNet). We first converted the mouse genes into human orthologs. Then, we found the intersecting genes between mouse tissue/cell expression profiles and human tissue/cell expression profiles. Limiting the input of human tissue RNA-seq profiles to the intersecting genes, we trained a CCN classifier with all the human tissue/cell expression profiles. The parameters used for the function “broadClass_train” in the package cancerCellNet are in Additional file 1: Table S1. We randomly sampled 75 samples from each tissue category in mouse tissue/cell data and applied the classifier on those samples to assess performance.

### Cross-species classifier benchmarking

The same set of training and validation data in the “Method” section “Cross-Species Assessment” was used to comprehensively benchmark various different classification methods. The specific training parameters for the 16 cross-species classifiers are recorded in Additional file 4: Table S4. In brief, we used the same data transformation and feature selection described in the “Method” sections “Rank-based random forest classifier” and “Gene pair-based KNN and SVM” to construct various gene rank and gene pair-based classifiers. For “ComBat+quantileNorm” data transformation, we followed the procedure described in Salvadores et al. [16]. We first performed quantile normalization of both human and mouse expression profiles using R package preprocessCore [46] and then performed ComBat batch corrections (with 2 batch labels of humans and mouse) using R package sva [47]. For “quantileNorm” data transformation, we only performed quantile normalization without batch correction.

### Cross-technology assessment

To assess the performance of CCN in applications to microarray data, we gathered 6,219 patient tumor microarray profiles across 12 different cancer types from approximately 75 different studies (Additional file 5: Table S5). We found the intersecting genes between the microarray profiles and TCGA patient RNA-seq profiles. Limiting the input of RNA-seq profiles to the intersecting genes, we created a CCN classifier with all the TCGA patient profiles using parameters for the function “broadClass_train” listed in Additional file 1: Table S1. After the microarray specific classifier was trained, we randomly sampled 60 microarray patient samples from each cancer category and applied CCN classifier on them as an assessment of the cross-technology performance.

### Comparison of selected gene pairs and random gene pairs

We performed 20 cross-validations (2/3 training data, 1/3 validation data) to compare the performance of CCN using selected gene pairs and randomly selected gene pairs. To ensure a fair comparison, at each cross-validation both methods of CCN were trained and validated using the same data, and the same CCN training parameters (nTopGenes = 30, nTopGenePairs = 75). Within each cross-validation, new sets of gene pairs were selected for both types of a classifier. We first selected 30 upregulated, 30 downregulated, and 30 invariant genes for each cancer category using CCN training pipeline for both types of classifiers. For normal CCN classifier, 75 gene pairs were selected for each cancer category. For the random gene pair CCN classifier, we computed all gene pairs using selected genes for each cancer category and combined them together. From the pool of gene pairs, we randomly selected 1650 gene pairs (the same number of gene pairs as 75 per category).

### Rank-based random forest classifier

We first ranked the gene expression per sample in ascending order so that the most lowly expressed gene was assigned as 1, and the most highly expressed gene was assigned a rank of the total number of genes. Then, we further selected the top 30 upregulated genes, 30 downregulated genes, and 30 invariant genes (30+30+30 genes) based on their ranks using template matching and constructed a random forest classifier. The rank-based random forest classifier was validated using tumor microarray data (Additional file 5: Table S5).

### Gene pair-based KNN and SVM

Using the gene pairs that were selected by the microarray CCN classifier, we transformed the TCGA training data. Then, we trained KNN classifier (Python package Scikit-learn [48]) and SVM classifier (Python package Scikit-learn [48]) and validated them using tumor microarray data (Additional file 5: Table S5).

### Training and validating scRNA-seq classifier

We extracted labeled human melanoma and glioblastoma scRNA-seq expression profiles [49, 50] and compiled the two datasets excluding 3 cell types T.CD4, T.CD8, and myeloid due to a low number of cells for training. Sixty cells from each of the 11 cell types were sampled for training a scRNA-seq classifier. The parameters for training a general scRNA-seq classifier using the function “broadClass_train” are in Additional file 1: Table S1. 25 cells from each of the 11 cell types from the held-out data were selected to assess the single-cell classifier. Using maximization of average macro F1 measure, we selected the decision threshold of 0.255. The gene pairs that were selected to construct the classifier are in Additional file 2: Table S2. To assess the cross-technology capability of applying scRNA-seq classifier to bulk RNA-seq, we extracted 305 normal human cell expression profiles spanning 4 purified cell types (B cells, endothelial cells, monocyte/macrophage, fibroblast) from data curated in the “Method” section “Cross-Species Assessment”.

### Training subtype CancerCellNet

We found 11 cancer types (BRCA, COAD, ESCA, HNSC, KIRC, LGG, PAAD, UCEC, STAD, LUAD, LUSC) which have meaningful subtypes based on either histology or molecular profile and have sufficient samples to train a subtype classifier with high AUPRCs. We also included normal tissue samples from BRCA, COAD, HNSC, KIRC, and UCEC to create a normal tissue category in the construction of their subtype classifiers. Training samples were either labeled as a cancer subtype for the cancer of interest or as “Unknown” if they belong to other cancer types. Similar to general classifier training, CCN performed gene pair transformation and selected the most discriminate gene pairs for each cancer subtype. In addition to the gene pairs selected to discriminate cancer subtypes, CCN also performed general classification of all training data and appended the classification profiles of training data with gene pair binary matrix as additional features. The reason behind using general classification profile as additional features is that many general cancer types may share similar subtypes, and general classification profile could be important features to discriminate the general cancer type of interest from other cancer types before performing finer subtype classification. The specific parameters used to train individual subtype classifiers using “subClass_train” function of CancerCellNet package can be found in Additional file 1: Table S1 and the gene pairs are in Additional file 2: Table S2.

### Validating subtype CancerCellNet

Similar to validating a general class classifier, we randomly sampled 2/3 of all samples in each cancer subtype as training data and sampled an equal amount across subtypes in the 1/3 held-out data for assessing subtype classifiers. We repeated the process 20 times for a more comprehensive assessment of subtype classifiers.

### Classifying query data into subtypes

We assigned subtype to query sample if the query sample has CCN score higher than the decision threshold. The table of decision threshold for subtype classifiers is in Additional file 3: Table S3. If no CCN scores exceed the decision threshold in any subtype or if the highest CCN score is in Unknown category, then we assigned that sample as Unknown. Analysis was performed in R and visualizations were generated with the ComplexHeatmap package [51].

### Cells culture, immunohistochemistry, and histomorphometry

Caov-4 (ATCC® HTB-76™), SK-OV-3(ATCC® HTB-77™), RT4 (ATCC® HTB-2™), and NCCIT(ATCC® CRL-2073™) cell lines were purchased from ATCC. HEC-59 (C0026001) and A2780 (93112519-1VL) were obtained from Addexbio Technologies and Sigma-Aldrich. Vcap and PC-3. SK-OV-3, Vcap, and RT4 were cultured in Dulbecco’s modified Eagle medium (DMEM, high glucose, 11960069, Gibco) with 1% penicillin-streptomycin-glutamine (10378016, Life Technologies); Caov-4, PC-3, NCCIT, and A2780 were cultured using RPMI-1640 medium (11875093, Gibco) while HEC-59 was in Iscove’s modified Dulbecco’s medium (IMDM, 12440053, Gibco). Both media were supplemented with 1% penicillin-streptomycin (15140122, Gibco). All mediums included 10% fetal bovine serum (FBS).

Cells cultured in a 48-well plate were washed twice with PBS and fixed in 10% buffered formalin for 24 h at 4°C. Immunostaining was performed using a standard protocol. Cells were incubated with primary antibodies to goat HOXB6 (10 μg/mL, PA5-37867, Invitrogen), mouse WT1 (10 μg/mL, MA1-46028, Invitrogen), rabbit PPARG (1:50, ABN1445, Millipore), mouse FOLH1 (10 μg/mL, UM570025, Origene), and rabbit LIN28A (1:50, #3978, Cell Signaling) in Antibody Diluent (S080981-2, DAKO), at 4 °C overnight followed with three 5 min washes in TBST. The slides were then incubated with secondary antibodies conjugated with fluorescence at room temperature for 1 h while avoiding light followed with three 5 min washes in TBST and nuclear stained with mounting medium containing DAPI. Images were captured by Nikon EcLipse Ti-S, DS-U3, and DS-Qi2.

Histomorphometry was performed using ImageJ (Version 2.0.0-rc-69/1.52i). % N.positive cells was calculated by the percentage of the number of positive stained cells divided by the number of DAPI-positive nucleus within three of randomly chosen areas. The data were expressed as means ± SD.

### Tumor purity analysis

We used the R package ESTIMATE [52] to calculate the ESTIMATE scores from TCGA tumor expression profiles that we used as training data for CCN classifier. To calculate tumor purity, we used the equation described in Yoshihara et al. [52]:

$$ \mathrm{Tumour}\ \mathrm{purity}=\cos \left(0.6049872018+0.0001467884\times \mathrm{ESTIMATE}\ \mathrm{score}\right) $$

### Extracting citation counts

We used the R package RISmed [53] to extract the number of citations for each cell line through query search of “*cell line name*[Text Word] AND *cancer*[Text Word]” on PubMed. The citation counts were normalized by dividing the citation counts with the number of years since first documented.

$$ \mathrm{Normalized}\ \mathrm{citation}\ \mathrm{counts}=\frac{\mathrm{citation}\ \mathrm{counts}}{\#\mathrm{years}\ \mathrm{since}\ \mathrm{first}\ \mathrm{documented}} $$

### GRN construction and GRN Status

Gene regulatory network (GRN) construction was extended from our previous method [54]. Eighty samples per cancer type were randomly sampled and normalized through downsampling as training data for the CLR GRN construction algorithm. Cancer type-specific GRNs were identified by determining the differentially expressed genes per each cancer type and extracting the subnetwork using those genes.

To extend the original GRN status algorithm [54] across different platforms and species, we devised a rank-based GRN status algorithm. Like the original GRN status, rank-based GRN status is a metric of assessing the similarity of cancer type-specific GRN between training data in the cancer type of interest and query samples. Hence, high GRN status represents a high level of establishment or similarity of the cancer-specific GRN in the query sample compared to those of the training data. Expression profiles of training data were first ranked using the same method described in the “Method” section “Rank-based random forest classifier”. Cancer type-specific mean and standard deviation of every gene’s rank expression were learned from training data. The modified *Z*-score values for genes within cancer type-specific GRN were calculated for query sample’s rank expression profiles to quantify the dissimilarity between query sample’s cancer type-specific GRN and that of the reference training data:

$$ \mathrm{Zscore}{\left(\mathrm{gene}\ \mathrm{i}\right)}_{\mathrm{mod}}=\left\{\begin{array}{c}0,\mathrm{if}\ \mathrm{Zscore}\ \mathrm{is}\ \mathrm{positive}\ \mathrm{and}\ \mathrm{the}\ \mathrm{gene}\ \mathrm{is}\ \mathrm{found}\ \mathrm{to}\ \mathrm{be}\ \mathrm{upregulated}\\ {}0,\mathrm{if}\ \mathrm{Zscore}\ \mathrm{is}\ \mathrm{negative}\ \mathrm{and}\ \mathrm{the}\ \mathrm{gene}\ \mathrm{is}\ \mathrm{found}\ \mathrm{to}\ \mathrm{be}\ \mathrm{downregulated}\\ {}\mathrm{abs}\left(\mathrm{Zscore}\right),\mathrm{otherwise}\ \end{array}\right. $$

If a gene in the cancer type-specific GRN is found to be upregulated in the specific cancer type relative to other cancer types and if the ranking of the query sample’s gene is equal to or greater than the mean ranking of the gene in the target training sample, then we would consider query sample’s gene to be similar to that of training sample. As a result of similarity, we assign that gene of a modified *Z*-score of 0. The same principle applies to cases where the gene is downregulated in cancer-specific subnetwork. Otherwise, the modified *Z*-score is the same as the absolute value of *Z*-score.

GRN status for query sample is calculated as the weighted mean of the (1000 − *Zscore*(*gene i*)_*mod*_) across genes in cancer type-specific GRN. 1000 is an arbitrary large number, and larger dissimilarity between query’s cancer type-specific GRN leads to high *Z*-scores for the GRN genes and low GRN status.

$$ {\displaystyle \begin{array}{c}\mathrm{RGS}={\sum}_{\mathrm{i}=1}^{\mathrm{n}}\left(1000-\mathrm{Zscore}{\left(\mathrm{gene}\ \mathrm{i}\right)}_{\mathrm{mod}}\right)\mathrm{weigh}{\mathrm{t}}_{\mathrm{gene}\ \mathrm{i}}\\ {}\mathrm{GRN}\ \mathrm{Status}=\frac{\mathrm{RGS}}{\sum_{\mathrm{i}=1}^{\mathrm{n}}\mathrm{weigh}{\mathrm{t}}_{\mathrm{gene}\ \mathrm{i}}}\end{array}} $$

The weight of individual genes in the cancer-specific network is determined by the importance of the gene in the CCN classifier. Finally, the GRN status gets normalized with respect to the GRN status of the cancer type of interest and the cancer type with the lowest mean GRN status.

$$ \mathrm{Normalized}\ \mathrm{GRN}\ \mathrm{s}\mathrm{tat}\mathrm{u}\mathrm{s}=\frac{\mathrm{GRN}\ \mathrm{s}\mathrm{tat}\mathrm{u}{\mathrm{s}}_{\mathrm{query}}-\mathrm{avg}\left(\mathrm{GRN}\ \mathrm{s}\mathrm{tat}\mathrm{u}{\mathrm{s}}_{\min\ \mathrm{cancer}}\right)}{\mathrm{avg}\left(\mathrm{GRN}\ \mathrm{s}\mathrm{tat}{\mathrm{us}}_{\mathrm{cancer}\ \mathrm{type}\ \mathrm{interest}}\right)-\mathrm{avg}\left(\mathrm{GRN}\ \mathrm{s}\mathrm{tat}\mathrm{u}{\mathrm{s}}_{\min\ \mathrm{cancer}}\right)} $$

“min cancer” represents the cancer type where its training data have the lowest mean GRN status in the cancer type of interest, and *avg*(*GRN status*_*min cancer*_) represents the lowest average GRN status in the cancer type of interest. *avg*(*GRN status*_*cancer type interest*_) represents the average GRN status of the cancer type of interest in the training data.

## Results

### CancerCellNet classifies samples accurately across species and technologies

**Fig. 1:**
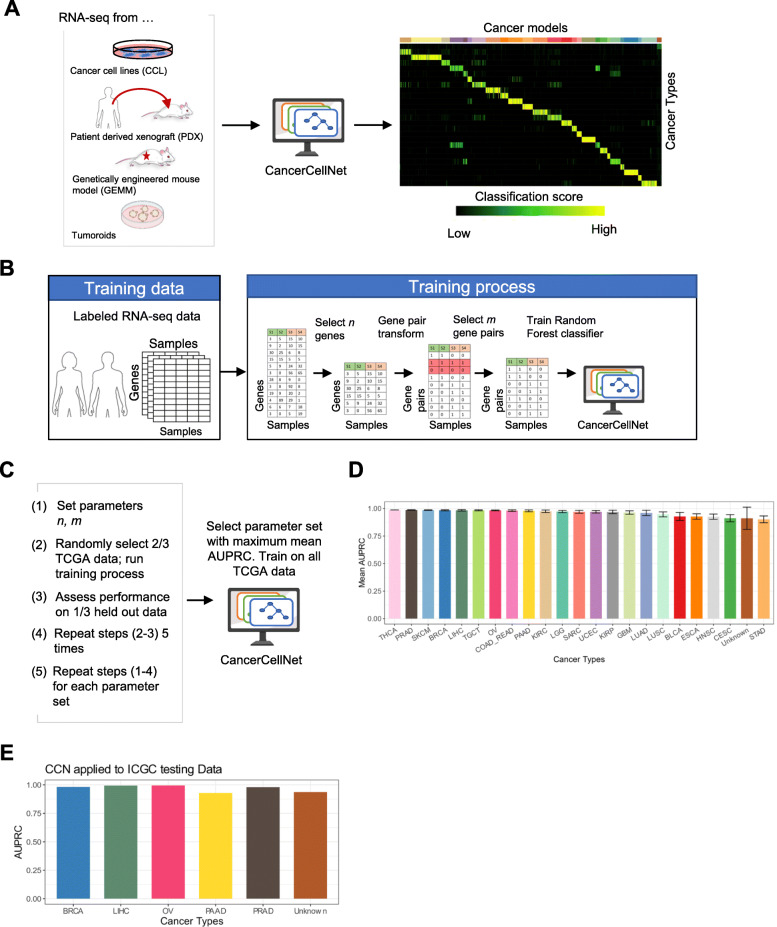
CancerCellNet (CCN) workflow, training, and performance. **a** Schematic of CCN usage. CCN was designed to assess and compare the expression profiles of cancer models such as CCLs, PDXs, GEMMs, and tumoroids with native patient tumors. To use trained classifier, CCN inputs the query samples (e.g. expression profiles from CCLs, PDXs, GEMMs, tumoroids) and generates a classification profile for the query samples. The column names of the classification heatmap represent sample annotation and the row names of the classification heatmap represent different cancer types. Each grid is colored from black to yellow representing the lowest classification score (e.g. 0) to highest classification score (e.g. 1). **b** Schematic of CCN training process. CCN uses patient tumor expression profiles of 22 different cancer types from TCGA as training data. First, CCN identifies *n* genes that are upregulated, *n* that are downregulated, and *n* that are relatively invariant in each tumor type versus all of the others. Then, CCN performs a pair transform on these genes and subsequently selects the most discriminative set of *m* gene pairs for each cancer type as features (or predictors) for the random forest classifier. Lastly, CCN trains a multi-class random forest classifier using gene-pair transformed training data. **c** Parameter optimization strategy. Five cross-validations of each parameter set in which 2/3 of TCGA data was used to train and 1/3 to validate was used to search for the values of *n* and *m* that maximized performance of the classifier as measured by area under the precision recall curve (AUPRC). **d** Mean and standard deviation of classifiers based on 50 cross-validations with the optimal parameter set. **e** AUPRCs of the final CCN classifier when applied to independent patient tumor data from ICGC

Previously, we had developed a computational tool using the random forest classification method to measure the similarity of engineered cell populations to their in vivo counterparts based on transcriptional profiles [45, 54]. More recently, we elaborated on this approach to classify single-cell RNA-seq data in a manner that allows for cross-platform and cross-species analysis [23]. Here, we used an analogous approach to build a platform that would allow us to quantitatively compare cancer models to naturally occurring patient tumors (Fig. 1a). In brief, we used TCGA RNA-seq expression data from 22 solid tumor types to train a top-pair multi-class random forest classifier (Fig. 1b). We combined training data from rectal adenocarcinoma (READ) and colon adenocarcinoma (COAD) into one COAD_READ category because READ and COAD are considered to be virtually indistinguishable at a molecular level [55]. We included an “Unknown” category trained using randomly shuffled gene pair profiles generated from the training data of 22 tumor types to identify query samples that are not reflective of any of the training data. To estimate the performance of CCN and how it is impacted by parameter variation, we performed a parameter sweep with 5-fold 2/3 cross-validation strategy (i.e., 2/3 of the data sampled across each cancer type was used to train, 1/3 was used to validate) (Fig. 1c). The performance of CCN, as measured by the mean area under the precision recall curve (AUPRC), did not fall below 0.945 and remained relatively stable across parameter sets (Additional file 6: Fig. S1A). The optimal parameters resulted in 1979 features. The mean AUPRCs exceeded 0.95 in most tumor types with this optimal parameter set (Fig. 1d, Additional file 6: Fig. S1B). The AUPRCs of CCN applied to independent RNA-seq data from 725 tumors across five tumor types from the International Cancer Genome Consortium (ICGC) [56] ranged from 0.93 to 0.99, supporting the notion that the platform is able to accurately classify tumor samples from diverse sources (Fig. 1e).

As one of the central aims of our study is to compare distinct cancer models, including GEMMs, our method needed to be able to classify samples from mouse and human samples equivalently. We used the Top-Pair transform [23] to achieve this, and we tested the feasibility of this approach by assessing the performance of a normal (i.e. non-tumor) cell and tissue classifier trained on human data and applied to mouse samples. Consistent with prior applications [23], we found that the cross-species classifier performed well, achieving the mean AUPRC of 0.97 when applied to mouse data (Additional file 6: Fig. S1C).

Since cross-species classification is relatively new in the application of assessing cancer models, we performed an exhaustive benchmark of 16 potential classification methods through different combinations of classification algorithms (Random Forest, SVM, KNN), data transformations (gene pairs, gene rank, quantile normalization, ComBat corrected quantile normalization), and feature selection strategies (selection of genes/gene pairs, none, random gene pairs generated from selected genes). For the comparison, because other classification methods do not have Unknown category, the AUPRC for Unknown category in CCN was removed. We found that 13 methods had mean AUPRCs greater than 0.95 (Additional file 6: Fig. S1D). CCN achieved the highest mean AUPRC of 0.98, had the lowest standard deviation in AUPRC of 0.014, and was the only classification method that had an AUPRC above 0.95 for all cell types suggesting that it is at least as good as other methods.

We also performed several benchmarking analyses to assess how transformation methods, classification algorithms, and feature selection strategies perform in terms of cross-platform classification of tumor data. To test gene pair transformation with an alternative platform-agnostic feature engineering method, we compared the performance of random forest classifiers trained using gene pairs and random forest classifiers trained using template matching [24] selected gene ranks (i.e. gene expression ranked in ascending order). The two classifiers performed similarly in terms of cross-platform (i.e. classifier trained using RNA-seq training data and applied to microarray query) performance with gene pair-based classifier achieving mean AUPRC (without “Unknown”) of 0.93 and gene rank-based classifier achieving 0.92 (Additional file 6: Fig. S1E). These results indicate that despite the potential loss of information through binarization of features, gene pairs are just as capable of producing high-performing classifiers as using the ranked expression data. Next, we compared the performance of gene pair random forest with two other classification algorithms: SVM and KNN. Similar to our results for cross-species benchmark, gene pair-based random forest (mean AUPRC 0.93) outperforms gene pair-based KNN (mean AUPRC 0.92) and SVM (mean AUPRC 0.67) (Additional file 6: Fig. S1F). Lastly, to study the effectiveness of gene pair selection, which is a computationally demanding step in the training process, we compared the performance of a CCN classifier trained using gene pairs selected using a second round of template matching [24] versus a classifier trained with gene pairs selected randomly from the genes derived from the first round of template matching. In short, the average mean AUPRCs across 20 cross-validations between the selected gene pairs CCN classifier and random gene pair CCN classifier are 0.959 and 0.956, respectively (Additional file 6: Fig. S1G). Even though the random selection of gene pairs in the second round is as good as another round of template matching, we used the latter to retain a degree of consistency in the set of gene pairs derived for the classifiers.

To evaluate cancer models at a finer resolution, we also developed a random forest-based approach to perform tumor subtype classifications (Additional file 6: Fig. S2A). Several recent studies have also developed methods to systematically categorize pan-cancer CCLs into cancer subtypes [14–16]. Yu et al. [14] utilized the Nearest Template Prediction [57] to construct 9 different solid tumor subtype classifiers based on transcriptomic data. Najgebauer et al. [15] developed the platform CELLector that utilizes genomic alternations to categorize CCLs into tumor subtypes. Salvadores et al. [16] used transcriptomic and epigenomic data to build ridge regression models for predicating subtypes in 15 general cancer types. Here, we constructed 11 different cancer subtype classifiers based on the availability of subtype information [55, 58–68]. We also included non-cancerous, normal tissues as categories for several subtype classifiers when sufficient data was available: breast invasive carcinoma (BRCA), COAD_READ, head and neck squamous cell carcinoma (HNSC), kidney renal clear cell carcinoma (KIRC), and uterine corpus endometrial carcinoma (UCEC). The 11 subtype classifiers all achieved high overall average AUPRCs ranging from 0.80 to 0.99 (Additional file 6: Fig. S2B).

### Fidelity of cancer cell lines

**Fig. 2:**
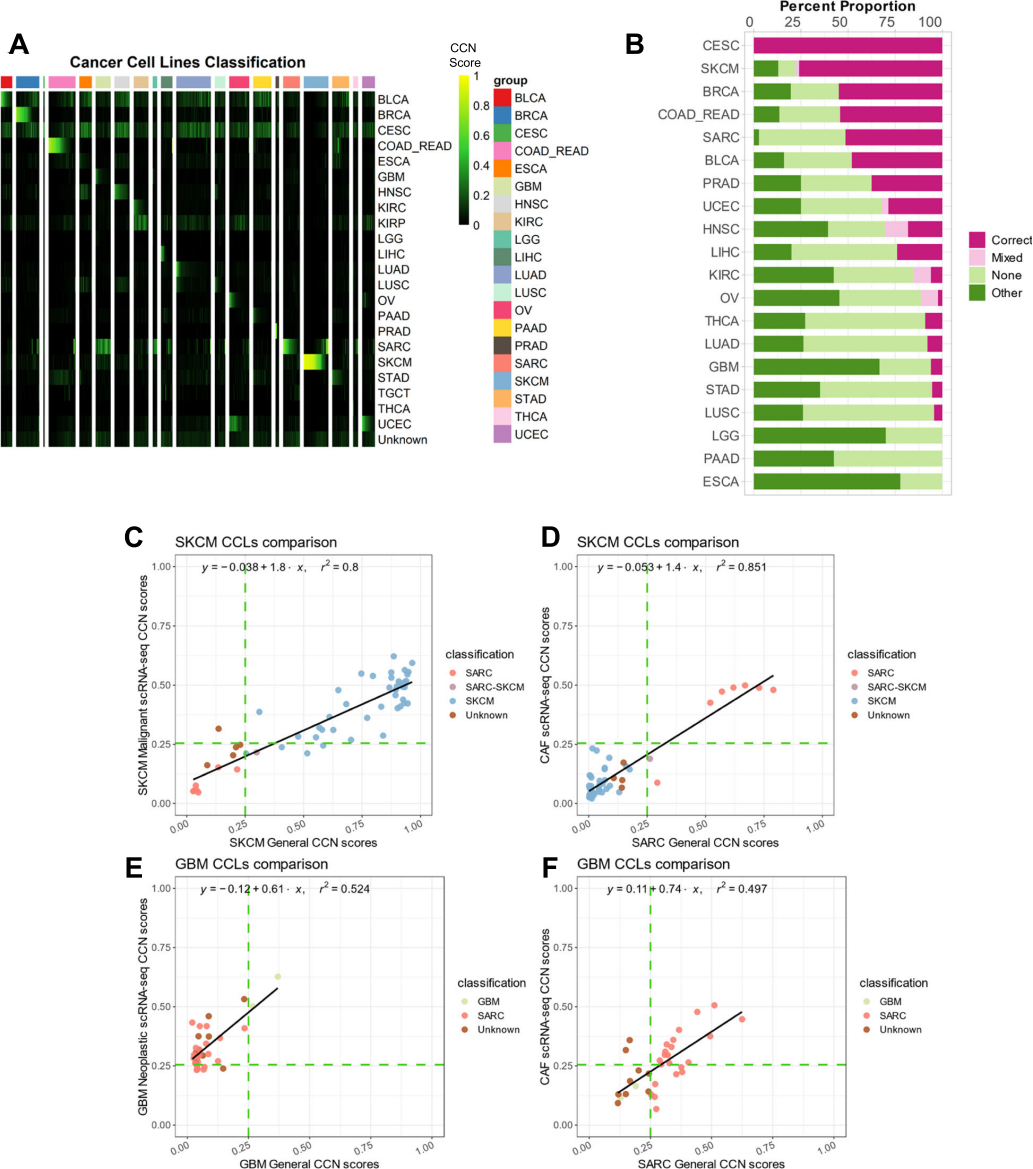
Evaluation of cancer cell lines. **a** General classification heatmap of CCLs extracted from CCLE. Column annotations of the heatmap represent the labeled cancer category of the CCLs given by CCLE and the row names of the heatmap represent different cancer categories. **b** Bar plot represents the proportions of each classification category in CCLs across cancer types ordered from the cancer types with the highest proportion of correct and correct mixed CCLs to lowest proportion. CCLs’ general classification profiles are categorized into 4 categories: correct (red), correct mixed (pink), no classification (light green), and other classification (dark green) based on the decision threshold of 0.25. **c** Comparison between SKCM general CCN scores from bulk RNA-seq classifier and SKCM malignant CCN scores from scRNA-seq classifier for SKCM CCLs. **d** Comparison between SARC general CCN scores from bulk RNA-seq classifier and CAF CCN scores from scRNA-seq classifier for SKCM CCLs. **e** Comparison between GBM general CCN scores from bulk RNA-seq classifier and GBM neoplastic CCN scores from scRNA-seq classifier for GBM CCLs. **f** Comparison between SARC general CCN scores and CAF CCN scores from scRNA-seq classifier for GBM CCLs. The green dotted lines indicate the decision threshold for scRNA-seq classifier and general classifier

Having validated the performance of CCN, we then used it to determine the fidelity of CCLs. We mined RNA-seq expression data of 657 different cell lines across 20 cancer types from the Cancer Cell Line Encyclopedia (CCLE) [69] and applied CCN to them, finding a wide classification range for cell lines of each tumor type (Fig. 2a, Additional file 7: Table S6). To verify the classification results, we applied CCN to expression profiles from CCLE generated through microarray expression profiling [28]. To ensure that CCN would function on microarray data, we tested it by applying a CCN classifier created to test microarray data to 720 expression profiles of 12 tumor types. The cross-platform CCN classifier performed well, based on the comparison to study-provided annotation, achieving a mean AUPRC (with Unknown) of 0.916 (Additional file 6: Fig. S3A). Next, we applied this cross-platform classifier to microarray expression profiles from CCLE (Additional file 6: Fig. S3B). From the classification results of 571 cell lines that have both RNA-seq and microarray expression profiles, we found a strong overall positive association between the classification scores from RNA-seq and those from microarray (Additional file 6: Fig. S3C). This comparison supports the notion that the classification scores for each cell line are not artifacts of profiling methodology. Moreover, this comparison shows that the scores are consistent between the times that the cell lines were first assayed by microarray expression profiling in 2012 and by RNA-seq in 2019. We also observed a high level of correlation between our analysis and the analysis done by Yu et al. [14] (Additional file 6: Fig. S3D), further validating the robustness of the CCN results.

Next, we assessed the extent to which CCN classifications agreed with their nominal tumor type of origin, which entailed translating quantitative CCN scores to classification labels. To achieve this, we selected a decision threshold that maximized the Macro F1 measure, the harmonic mean of precision and recall, across 50 cross-validations. Then, we annotated cell lines based on their CCN score profiles as follows. Cell lines with CCN scores > threshold for the tumor type of origin were annotated as “correct.” Cell lines with CCN scores > threshold in the tumor type of origin and at least one other tumor type were annotated as “mixed.” Cell lines with CCN scores > threshold for tumor types other than that of the cell line’s origin were annotated as “other.” Cell lines that did not receive a CCN score > threshold for any tumor type were annotated as “none” (Fig. 2b). We found that majority of cell lines originally annotated as breast invasive carcinoma (BRCA), cervical squamous cell carcinoma, and endocervical adenocarcinoma (CESC), skin cutaneous melanoma (SKCM), colorectal cancer (COAD_READ), and sarcoma (SARC) fell into the “correct” category (Fig. 2b). On the other hand, no esophageal carcinoma (ESCA), pancreatic adenocarcinoma (PAAD), or brain lower grade glioma (LGG) were classified as “correct,” demonstrating the need for more transcriptionally faithful cell lines that model those general cancer types.

There are several possible explanations for cell lines not receiving a “correct” classification. One possibility is that the sample was incorrectly labeled in the study from which we harvested the expression data. Consistent with this explanation, we found that colorectal cancer line NCI-H684 [70, 71], a cell line mislabeled as liver hepatocellular carcinoma (LIHC), was classified strongly as COAD_READ (Additional file 7: Table S6). Similarly, our findings indicate that COLO 741, a skin melanoma cell line historically mistaken to be of colon adenocarcinoma origin [70], was classified as SKCM (Additional file 7: Table S6). This finding agrees with those of Salvadores et al. [16]. Another possibility to explain low CCN score is that cell lines were derived from subtypes of tumors that are not well-represented in TCGA. To explore this hypothesis, we first performed tumor subtype classification on CCLs from 11 tumor types for which we had trained subtype classifiers (Additional file 8: Table S7). We reasoned that if a cell was a good model for a rarer subtype, then it would receive a poor general classification but a high classification for the subtype that it models well. Therefore, we counted the number of lines that fit this pattern. We found that of the 188 lines with no general classification, 25 (13%) were classified as a specific subtype, suggesting that derivation from rare subtypes is not the major contributor to the poor overall fidelity of CCLs.

Another potential contributor to low-scoring cell lines is intra-tumor stromal and immune cell impurity in the training data. If impurity were a confounder of CCN scoring, then we would expect a strong positive correlation between mean purity and mean CCN classification scores of CCLs per general tumor type. However, the Pearson correlation coefficient between the mean purity of general tumor type and mean CCN classification scores of CCLs in the corresponding general tumor type was low (0.14), suggesting that tumor purity is not a major contributor to the low CCN scores across CCLs (Additional file 6: Fig. S3E).

### Comparison of SKCM and GBM CCLs to scRNA-seq

To more directly assess the impact of intra-tumor heterogeneity in the training data on evaluating cell lines, we constructed a classifier using cell types found in human melanoma and glioblastoma scRNA-seq data [49, 50]. Previously, we have demonstrated the feasibility of using our classification approach on scRNA-seq data [23]. Our scRNA-seq classifier achieved a high average AUPRC (0.95) when applied to held-out data and high mean AUPRC (0.99) when applied to few purified bulk testing samples (Additional file 6: Fig. S4A-B). Comparing the CCN score from bulk RNA-seq general classifier and scRNA-seq classifier, we observed a high level of correlation (Pearson correlation of 0.89) between the SKCM CCN classification scores and scRNA-seq SKCM malignant CCN classification scores for SKCM cell lines (Fig. 2c, Additional file 6: Fig. S4C). Of the 41 SKCM cell lines that were classified as SKCM by the bulk classifier, 37 were also classified as SKCM malignant cells by the scRNA-seq classifier. Interestingly, we also observed a high correlation between the SARC CCN classification score and scRNA-seq cancer-associated fibroblast (CAF) CCN classification scores (Pearson correlation of 0.92) (Fig. 2d). Six of the seven SKCM cell lines that had been classified as exclusively SARC by CCN were classified as CAF by the scRNA-seq classifier (Fig. 2d, Additional file 6: Fig. S4C). This suggests the possibility that these cell lines were derived from CAF or other mesenchymal populations, or that they have acquired a mesenchymal character through their derivation. The high level of agreement between scRNA-seq and bulk RNA-seq classification results shows that heterogeneity in the training data of general CCN classifier has little impact in the classification of SKCM cell lines.

In contrast, we observed a weaker correlation between GBM CCN classification scores and scRNA-seq GBM neoplastic CCN classification scores (Pearson correlation of 0.72) for GBM cell lines (Fig. 2e, Additional file 6: Fig. S4D). Of the 31 GBM lines that were not classified as GBM with CCN, 25 were classified as GBM neoplastic cells with the scRNA-seq classifier. Among the 22 GBM lines that were classified as SARC with CCN, 15 cell lines were classified as CAF (Fig. 2f), 10 of which were classified as both GBM neoplastic and CAF in the scRNA-seq classifier. Similar to the situation with SKCM lines that classify as CAF, this result is consistent with the possibility that some GBM lines classified as SARC by CCN could be derived from mesenchymal subtypes exhibiting both strong mesenchymal signatures and glioblastoma signatures or that they have acquired a mesenchymal character through their derivation. The lower level of agreement between scRNA-seq and bulk RNA-seq classification results for GBM models suggests that the heterogeneity of glioblastomas [72] can impact the classification of GBM cell lines, and that the use of scRNA-seq classifier can resolve this deficiency.

### Immunofluorescence confirmation of CCN predictions

To experimentally explore some of our computational analyses, we performed immunofluorescence on three cell lines that were not classified as their labeled categories: the ovarian cancer line SK-OV-3 had a high UCEC CCN score (0.246), the ovarian cancer line A2780 had a high Testicular Germ Cell Tumors (TGCT) CCN score (0.327), and the prostate cancer line PC-3 had a high bladder cancer (BLCA) score (0.307) (Additional file 7: Table S6). We reasoned that if SK-OV-3, A2780, and PC-3 were classified most strongly as UCEC, TGCT, and BLCA, respectively, then they would express proteins that are indicative of these cancer types.

**Fig. 3:**
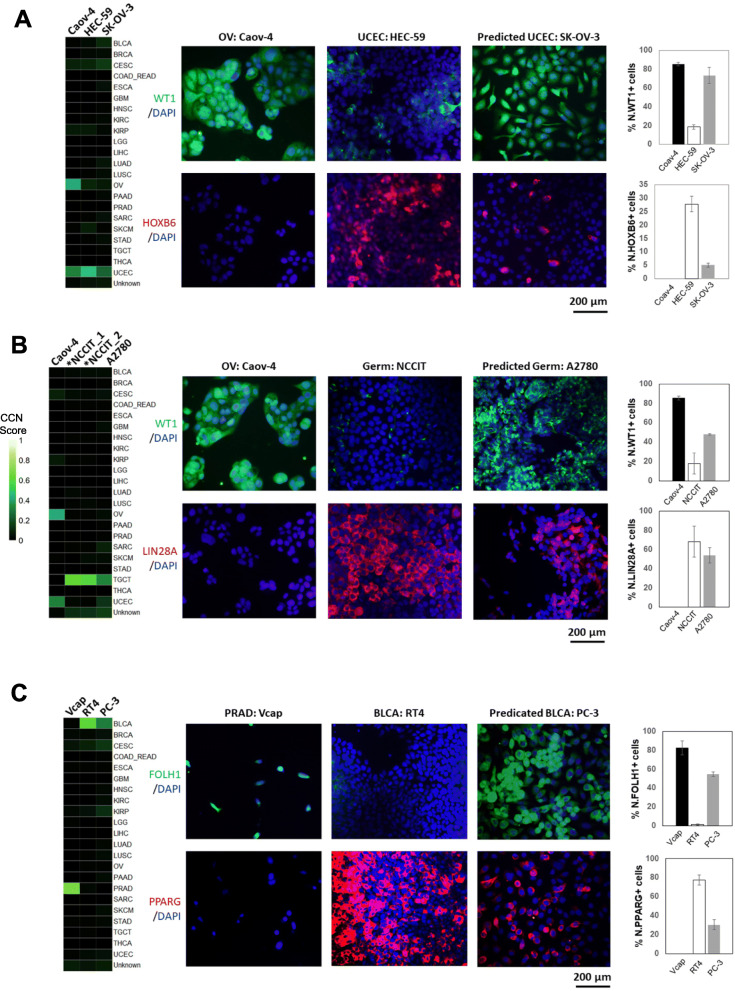
Immunofluorescence of selected cell lines. **a** Classification profiles (left) and IF expression (middle) of Caov-4 (OV-positive control), HEC-59 (UCEC-positive control), and SK-OV-3 for WT1 (OV biomarker) and HOXB6 (uterine biomarker). The rightmost bar plots quantify the average percentage of positive cells for WT1 (top-right) and HOXB6 (bottom-right). **b** Classification profiles (left) and IF expression (middle) of Caov-4, NCCIT (germ cell tumor-positive control), and A2780 for WT1 and LIN28A (germ cell tumor biomarker). Classification was performed on replicates of NCCIT RNA-seq profiles. The rightmost bar plots quantify the average percentage of positive cells for WT1 (top-right) and LIN28A (bottom-right). **c** Classification profiles (left) and IF expression (middle) of Vcap (PRAD positive control), RT4 (BLCA-positive control), and PC-3 for FOLH1 (prostate biomarker) and PPARG (urothelial biomarker). The rightmost bar plots quantify the average percentage of positive cells for FOLH1 (top-right) and PPARG (bottom-right)

First, we measured the expression of the uterine-associated transcription factor HOXB6 [73, 74], and the UCEC serous ovarian tumor biomarker WT1 [75] in SK-OV-3, in the OV cell line Caov-4, and in the UCEC cell line HEC-59. We chose Caov-4 as our positive control for OV biomarker expression because it was determined by our analysis and others [10, 14] to be a good model of OV. Likewise, we chose HEC-59 to be a positive control for UCEC. We found that SK-OV-3 has a small percentage (5%) of cells that expressed the uterine marker HOXB6 and a large proportion (73%) of cells that expressed WT1 (Fig. 3a). In contrast, no Caov-4 cells expressed HOXB6, whereas 85% of cells expressed WT1. This suggests that SK-OV-3 exhibits both biomarkers of ovarian tumor and uterine tissue. From our computational analysis and experimental validation, SK-OV-3 is most likely an endometrioid subtype of ovarian cancer. This result is also consistent with prior classification of SK-OV-3 [76], and the fact that SK-OV-3 lacks p53 mutations, which is prevalent in high-grade serous ovarian cancer [77], and it harbors an endometrioid-associated mutation in ARID1A [10, 76, 78]. Next, we measured the expression of markers of OV and germ cell cancers (LIN28A [79]) in the OV-annotated cell line A2780, which received a high TCGT CCN score comparable to those of human embryonic carcinoma cells, NCCIT [29] (Fig. 3b). Fifty-four percent of A2780 and 66% of NCCIT cells expressed LIN28A, whereas it was not detected in Caov-4 (Fig. 3b). The OV marker WT1 was also expressed in fewer A2780 cells as compared to Caov-4 (48% vs 85%), which suggests that A2780 could be a germ cell-derived ovarian tumor. Taken together, our results suggest that SK-OV-3 and A2780 could represent OV subtypes that are not well represented in TCGA training data, which resulted in a low OV score and higher CCN score in other categories.

Lastly, we examined PC-3, annotated as a PRAD cell line but classified to be most similar to BLCA. We found that 30% of the PC-3 cells expressed PPARG, a contributor to urothelial differentiation [80] that is not detected in the PRAD Vcap cell line but is highly expressed in the BLCA RT4 cell line (Fig. 3c). PC-3 cells also expressed the PRAD biomarker FOLH1 [81] suggesting that PC-3 has a PRAD origin and gained urothelial or luminal characteristics through the derivation process. In short, our limited experimental data support the CCN classification results.

### Subtype classification of cancer cell lines

**Fig. 4:**
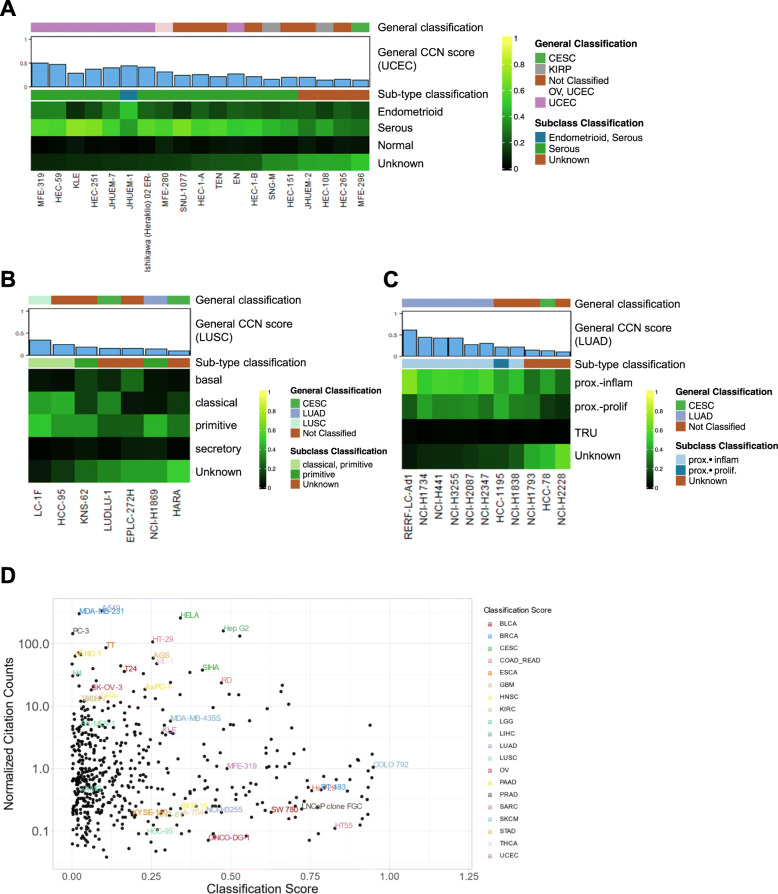
Subtype classification of CCLs and CCL prevalence. The heatmap visualizations of subtype classification for **a** UCEC CCLs, **b** LUSC CCLs, and **c** LUAD CCLs. Only samples with general CCN scores > 0.1 in their nominal tumor type are displayed. **d** Comparison of normalized citation counts and general CCN classification scores of CCLs. Labeled cell lines either have the highest CCN classification score in their labeled cancer category or highest normalized citation count. Each citation count was normalized by a number of years since first documented on PubMed

Next, we explored the subtype classification of CCLs from three general tumor types in more depth. We focused our subtype visualization (Fig. 4a–c) on CCL models with general CCN score above 0.1 in their nominal cancer type as this allowed us to analyze those models that fell modestly below the general threshold but were classified as a specific sub-type (Additional file 7: Table S6, Additional file 8: Table S7). We focused first on UCEC. The histologically defined subtypes of UCEC, endometrioid and serous, differ in prevalence, molecular properties, prognosis, and treatment. For instance, the endometrioid subtype, which accounts for approximately 80% of uterine cancers, retains estrogen receptor and progesterone receptor status and is responsive towards progestin therapy [82, 83]. Serous, a more aggressive subtype, is characterized by the loss of estrogen and progesterone receptor and is not responsive to progestin therapy [82, 83]. CCN classified the majority of the UCEC cell lines as serous except for JHUEM-1 which is classified as mixed, with similarities to both endometrioid and serous (Fig. 4a). The preponderance CCLE lines of serous versus endometroid character may be due to properties of serous cancer cells that promote their in vitro propagation, such as upregulation of cell adhesion transcriptional programs [84]. Some of our subtype classification results are consistent with prior observations. For example, HEC-1A, HEC-1B, and KLE were previously characterized as type II endometrial cancer, which includes a serous histological subtype [85]. On the other hand, our subtype classification results contradict prior observations in at least one case. For instance, the Ishikawa cell line was derived from type I endometrial cancer (endometrioid histological subtype) [85, 86]; however, CCN classified a derivative of this line, Ishikawa 02 ER-, as serous. The high serous CCN score could result from a shift in phenotype of the line concomitant with its loss of estrogen receptor (ER) as this is a distinguishing feature of type II endometrial cancer (serous histological subtype) [82]. Taken together, these results indicate a need for more endometroid-like CCLs.

Next, we examined the subtype classification of lung squamous cell carcinoma (LUSC) and lung adenocarcinoma (LUAD) cell lines (Fig. 4b, c). All the LUSC lines with at least one subtype classification had an underlying primitive subtype classification. This is consistent either with the ease of deriving lines from tumors with a primitive character, or with a process by which cell line derivation promotes similarity to more primitive subtype, which is marked by increased cellular proliferation [60]. Some of our results are consistent with prior reports that have investigated the resemblance of some lines to LUSC subtypes. For example, HCC-95, previously characterized as classical [60, 87], had a maximum CCN score in the classical subtype (0.429). Similarly, LUDLU-1 and EPLC-272H, previously reported as classical [87] and basal [87] respectively, had maximal tumor subtype CCN scores for these subtypes (0.323 and 0.256) (Fig. 4b, Additional file 8: Table S7) despite being classified as Unknown. To place our results in the context of more recent work on subtyping CCLs, we compared our subtype results to two studies that made subtype predictions using the same LUSC subtype classification system. CCN generated high sub-type scores in agreement with 11/22 (45%) of the lines predicted by Yu et al. and 4/11 (36%) of the lines predicted by Salvadores et al. (Additional file 9: Table S8). The overall moderate levels of agreement could be explained by some combination of differences in methodologies, data type, and a loss of sub-type character upon line derivation.

Lastly, the LUAD cell lines that were classified as a subtype were either classified as proximal inflammation or proximal proliferation (Fig. 4c). RERF-LC-Ad1 had the highest general classification score and the highest proximal inflammation subtype classification score. Taken together, these subtype classification results suggest an absence of cell line models for basal and secretory LUSC and for the terminal respiratory unit (TRU) LUAD subtype.

### Cancer cell lines’ popularity and transcriptional fidelity

Finally, we sought to measure the extent to which cell line transcriptional fidelity related to model prevalence. We used the number of papers in which a model was mentioned, normalized by the number of years since the cell line was documented, as a rough approximation of model prevalence. To explore this relationship, we plotted the normalized citation count versus general classification score, labeling the highest cited and highest classified cell lines from each general tumor type (Fig. 4d). For most of the general tumor types, the highest cited cell line is not the highest classified cell line except for Hep G2, AGS, and ML-1, representing liver hepatocellular carcinoma (LIHC), stomach adenocarcinoma (STAD), and thyroid carcinoma (THCA), respectively. On the other hand, the general scores of the highest cited cell lines representing BLCA (T24), BRCA (MDA-MB-231), and PRAD (PC-3) fall below the classification threshold of 0.25. Notably, each of these tumor types has other lines with scores exceeding 0.5, which should be considered as more faithful transcriptional models when selecting lines for a study (Additional file 7: Table S6 and http://www.cahanlab.org/resources/cancerCellNet_results/).

### Evaluation of patient-derived xenografts

**Fig. 5:**
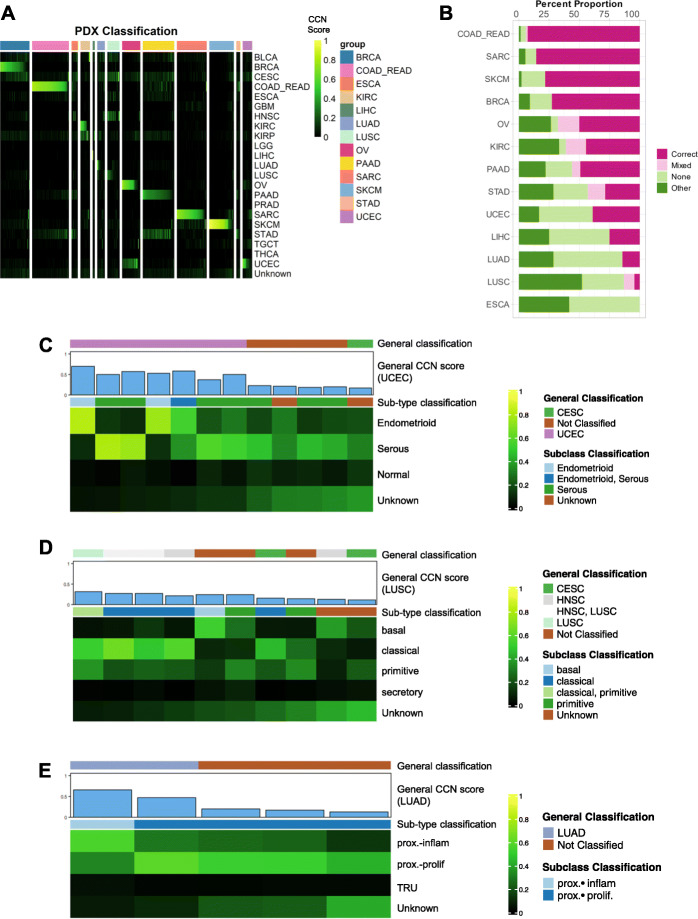
Evaluation of patient derived xenografts. **a** General classification heatmap of PDXs. Column annotations represent annotated cancer type of the PDXs and row names represent cancer categories. **b** Proportions of classification categories in PDXs across cancer types are visualized in the bar plot and ordered from the cancer type with the highest proportion of correct and mixed correct PDXs to the lowest. Subtype classification heatmaps of **c** UCEC PDXs, **d** LUSC PDXs, and **e** LUAD PDXs. Only samples with general CCN scores > 0.1 in their nominal tumor type are displayed

Next, we sought to evaluate a more recent class of cancer models: PDX. To do so, we subjected the RNA-seq expression profiles of 415 PDX models from 13 different cancer types generated previously [19] to CCN. Similar to the results of CCLs, the PDXs exhibited a wide range of classification scores (Fig. 5a, Additional file 10: Table S9). By categorizing the CCN scores of PDX based on the proportion of samples associated with each tumor type that were correctly classified, we found that SARC, SKCM, COAD_READ, and BRCA have a higher proportion of correctly classified PDX than those of other cancer categories (Fig. 5b). In contrast to CCLs, we found a higher proportion of correctly classified PDX in STAD, PAAD, and KIRC (Fig. 5b). However, similar to CCLs, no ESCA PDXs were classified as such. This held true when we performed subtype classification on PDX samples: none of the PDX in ESCA was classified as any of the ESCA subtypes (Additional file 11: Table S10). UCEC PDXs had both endometrioid subtypes, serous subtypes, and mixed subtypes, which provided a broader representation than CCLs (Fig. 5c). Several LUSC PDXs that were classified as a subtype were also classified as head and neck squamous cell carcinoma (HNSC) or mix HNSC and LUSC (Fig. 5d). This could be due to the similarity in expression profiles of basal and classical subtypes of HNSC and LUSC [60, 88], which is consistent with the observation that these PDXs were also subtyped as classical. No LUSC PDXs were classified as the secretory subtype. In contrast to LUAD CCLs, four of the five LUAD PDXs with a discernible sub-type were classified as proximal proliferation (Fig. 5e). On the other hand, similar to the CCLs, there were no TRU subtypes in the LUAD PDX cohort. In summary, we found that while individual PDXs can reach extremely high transcriptional fidelity for both general tumor types and subtypes, many PDXs were not classified as the general tumor type from which they originated.

### Evaluation of GEMMs

**Fig. 6:**
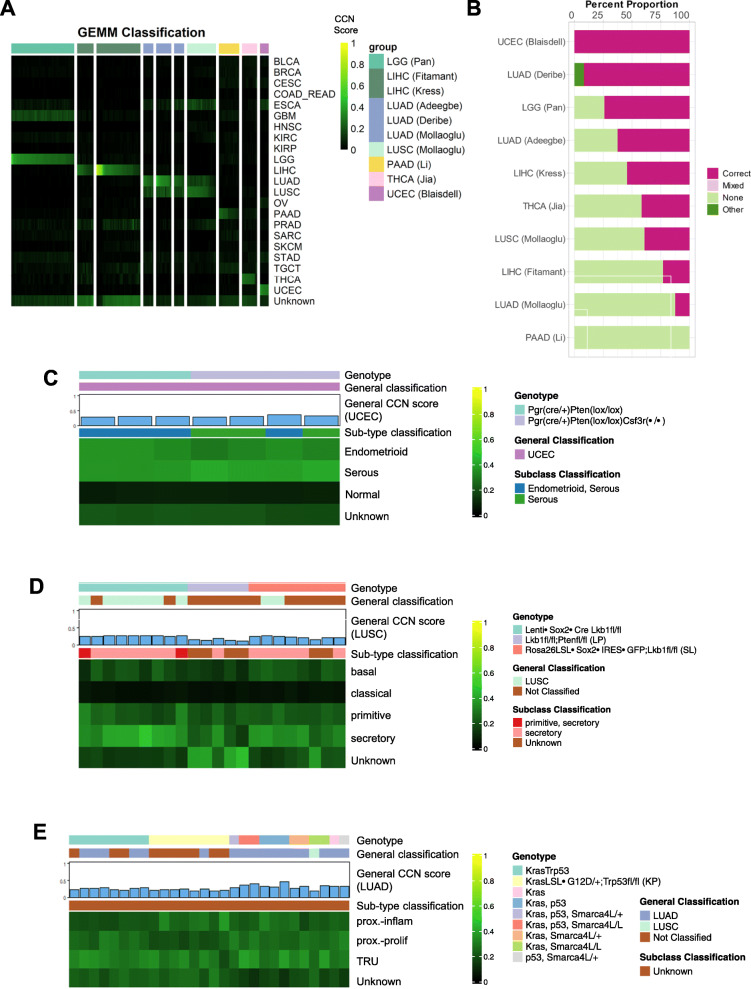
Evaluation of genetically engineered mouse models. **a** General classification heatmap of GEMMs. Column annotations represent annotated cancer type of the GEMMs, and row names represent cancer categories. **b** Proportions of classification categories in GEMMs across cancer types are visualized in the bar plot and ordered from the cancer type with the highest proportion of correct and mixed correct GEMMs to the lowest. Subtype classification heatmap of **c** UCEC GEMMs, **d** LUSC GEMMs, and **e** LUAD GEMMs. Only samples with general CCN scores > 0.1 in their nominal tumor type are displayed

Next, we used CCN to evaluate GEMMs of six general tumor types from nine studies for which expression data was publicly available [30–38]. As was true for CCLs and PDXs, GEMMs also had a wide range of CCN scores (Fig. 6a, Additional file 12: Table S11). We next categorized the CCN scores based on the proportion of samples associated with each tumor type that were correctly classified (Fig. 6b). In contrast to LGG CCLs, LGG GEMMs, generated by Nf1 mutations expressed in different neural progenitors in combination with Pten deletion [37], consistently were classified as LGG (Fig. 6a, b). The GEMM dataset included multiple replicates per model, which allowed us to examine intra-GEMM variability. Both at the level of CCN scores and at the level of categorization, GEMMs were invariant. For example, replicates of UCEC GEMMs driven by Prg(cre/+)Pten(lox/lox) received almost identical general CCN scores (Fig. 6c, Additional file 12: Table S11). GEMMs sharing genotypes across studies, such as LUAD GEMMs driven by Kras mutation and loss of p53 [30, 36, 38], also received similar general and subtype classification scores (Fig. 6a, b, e).

Next, we explored the extent to which genotype impacted subtype classification in UCEC, LUSC, and LUAD (Fig. 6c–e, Additional file 13: Table S12). Prg(cre/+)Pten(lox/lox) GEMMs had a mixed subtype classification of both serous and endometrioid, consistent with the fact that Pten loss occurs in both subtypes (albeit more frequently in endometrioid). We also analyzed Prg(cre/+)Pten(lox/lox)Csf3r-/- GEMMs. Polymorphonuclear neutrophils (PMNs), which play anti-tumor roles in endometrioid cancer progression, are depleted in these animals. Interestingly, Prg(cre/+)Pten(lox/lox)Csf3r-/- GEMMs had a serous subtype classification, which could be explained by differences in PMN involvement in endometrioid versus serous uterine tumor development that are reflected in the respective transcriptomes of the TCGA UCEC training data. We note that the tumor cells were sorted prior to RNA-seq, and thus, the shift in subtype classification is not due to contamination of GEMMs with non-tumor components. In short, this analysis supports the argument that tumor-cell extrinsic factors; in this case, a reduction in anti-tumor PMNs can shift the transcriptome of a GEMM so that it more closely resembles a serous rather than endometrioid subtype.

The LUSC GEMMs that we analyzed were Lkb1^fl/fl^ and they either overexpressed of Sox2 (via two distinct mechanisms) or were also Pten^fl/fl^ [36]. We note that the eight lenti-Sox2-Cre-infected, Lkb1^fl^/^fl^ and Rosa26LSL-Sox2-IRES-GFP, and Lkb1^fl^/^fl^ samples that classified as “Unknown” had LUSC CCN scores only modestly lower than the decision threshold (Fig. 6d) (mean CCN score = 0.217). Thirteen out of the 17 of the Sox2 GEMMs classified as the secretory subtype of LUSC. The consistency is not surprising given both models overexpress Sox2 and lose Lkb1. On the other hand, the Lkb1^fl/fl^ and Pten^fl/fl^ GEMMs had substantially lower general LUSC CCN scores, and our subtype classification indicated that this GEMM was mostly classified as “Unknown,” in contrast to prior reports suggesting that it is most similar to a basal subtype [89]. None of the three LUSC GEMMs has strong classical CCN scores. Most of the LUAD GEMMs, which were generated using various combinations of activating Kras mutation, loss of Trp53, and loss of Smarca4L [30, 36, 38], were correctly classified (Fig. 6e). Those that were not classified have modestly lower CCN scores than the decision threshold (mean CCN score = 0.214). There were no substantial differences in general or subtype classification across driver genotypes. Although the sub-type of all LUAD GEMMs was “Unknown,” the subtypes tended to have a mixture of high CCN proximal proliferation, proximal inflammation, and TRU scores. Taken together, this analysis suggests that there is a degree of similarity and perhaps plasticity between the primitive and secretory (but not basal or classical) subtypes of LUSC. On the other hand, while the LUAD GEMMs classify strongly as LUAD, they do not have a strong particular subtype classification—a result that does not vary by genotype.

### Evaluation of tumoroids

**Fig. 7:**
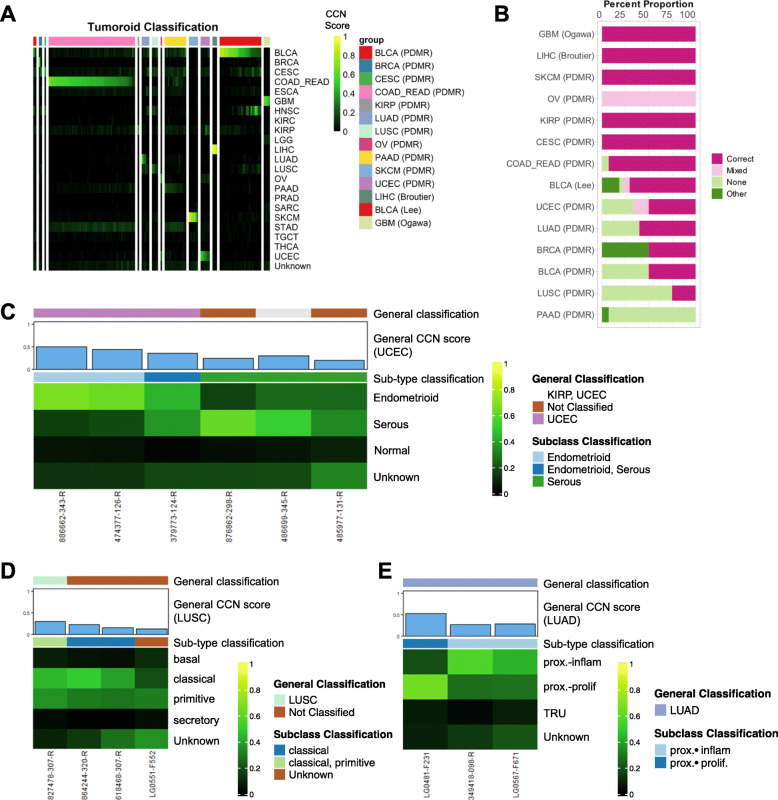
Evaluation of tumoroid models. **a** General classification heatmap of tumoroids. Column annotations represent annotated cancer type of the tumoroids, and row names represent cancer categories. **b** Proportions of classification categories in tumoroids across cancer types are visualized in the bar plot and ordered from the cancer type with the highest proportion of correct and mixed correct tumoroids to the lowest. Subtype classification heatmap of **c** UCEC tumoroids, **d** LUSC tumoroids, and **e** LUAD tumoroids. Only samples with general CCN scores > 0.1 in their nominal tumor type are displayed

Lastly, we used CCN to assess a relatively novel cancer model: tumoroids. We downloaded and assessed 131 distinct tumoroid expression profiles spanning 13 cancer categories from the NCI patient-derived models repository (PDMR) [39] and from three individual studies [40–42] (Fig. 7a, Additional file 14: Table S13). We note that several categories have three or fewer samples (BRCA, CESC, KIRP, OV, BLCA (PDMR), and LIHC). Among the cancer categories represented by more than three samples, only LUSC and PAAD have fewer than 50% classified as their annotated label (Fig. 7b). In contrast to GBM CCLs, all three induced pluripotent stem cell-derived GBM tumoroids [42] were classified as GBM with high CCN scores (mean = 0.53). To further characterize the tumoroids, we performed subtype classification on them (Additional file 15: Table S14). UCEC tumoroids from PDMR contain a wide range of subtypes with two endometrioid, two serous, and one mixed type (Fig. 7c). On the other hand, LUSC tumoroids appear to be predominantly of classical subtypes with one tumoroid classified as a mix between classical and primitive (Fig. 7d). Lastly, similar to the CCL and PDX counterparts, LUAD tumoroids are classified as proximal inflammatory and proximal proliferation with no tumoroids classified as TRU subtype (Fig. 7e).

### Comparison of CCLs, PDXs, GEMMs, and tumoroids

**Fig. 8:**
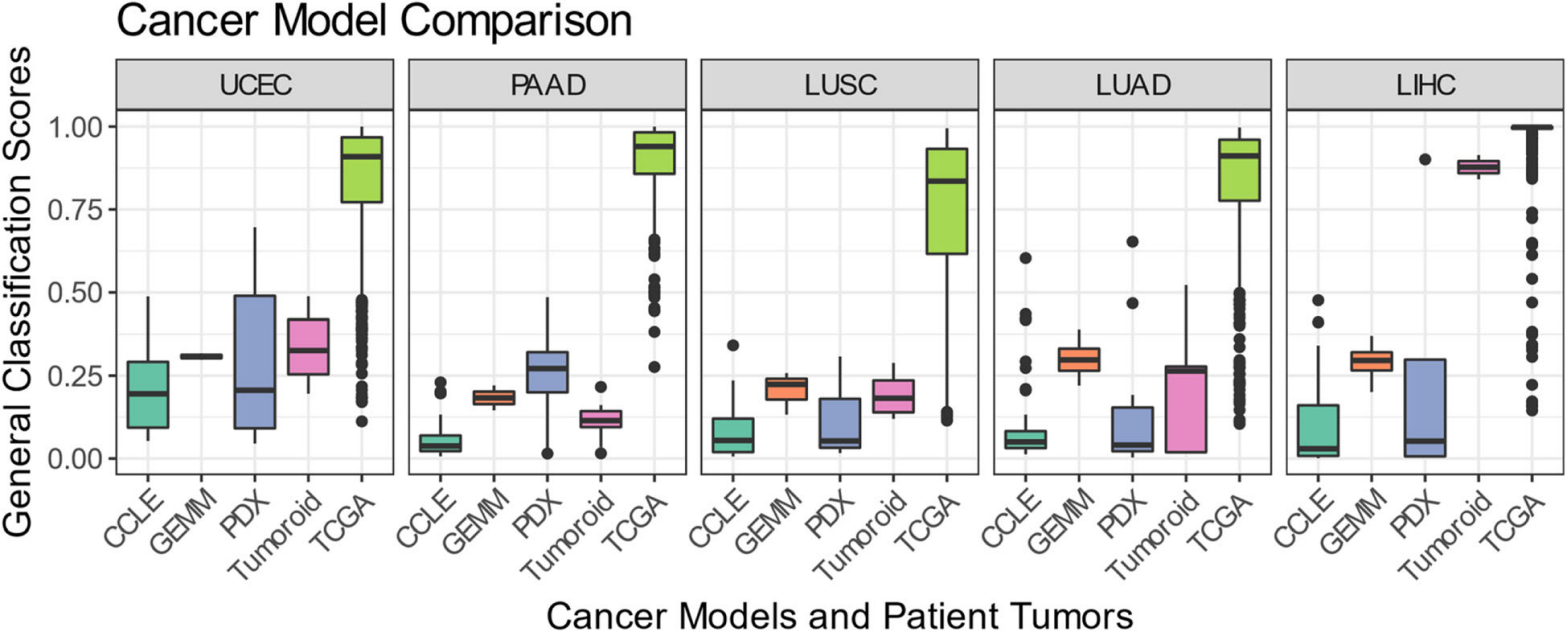
Comparison of CCLs, PDXs, GEMMs, and tumoroids. Box-and-whiskers plot comparing general CCN scores across CCLs, GEMMs, PDXs, and tumoroids in five general tumor types (UCEC, PAAD, LUSC, LUAD, LIHC)

Finally, we sought to estimate the comparative transcriptional fidelity of the four cancer model modalities. We compared the general CCN scores of each model on a per tumor type basis (Fig. 8). In the case of GEMMs, we used the mean classification score of all samples with shared genotypes. We also used the mean classification of technical replicates found in LIHC tumoroids [40]. We evaluated models based on both the maximum CCN score, as this represents the potential for a model class, and the median CCN score, as this indicates the current overall transcriptional fidelity of a model class. PDXs achieved the highest CCN scores in three (UCEC, PAAD, LUAD) out of the five cancer categories in which all four modalities were available (Fig. 8), despite having low median CCN scores. Notably, PDXs have a median CCN score above the 0.25 threshold in PAAD while none of the other three modalities have any samples above the threshold. In LIHC, the highest CCN score for PDX (0.9) is only slightly lower than the highest CCN score for tumoroid (0.91). This suggests that certain individual PDXs most closely mimic the transcriptional state of native patient tumors despite a portion of the PDXs having low CCN scores. Similarly, while the majority of the CCLs have low CCN scores, several lines achieve high transcriptional fidelity in LUSC, LUAD, and LIHC (Fig. 8). Collectively, GEMMs and tumoroids had the highest median CCN scores in four of the five model classes (LUSC and LUAD for GEMMs and UCEC and LIHC for tumoroids). Notably, both of the LIHC tumoroids achieved CCN scores on par with patient tumors (Fig. 8). In brief, this analysis indicates that PDXs and CCLs are heterogenous in terms of transcriptional fidelity, with a portion of the models highly mimicking native tumors and the majority of the models having low transcriptional fidelity (with the exception of PAAD for PDXs). On the other hand, GEMMs and tumoroids displayed a consistently high fidelity across different models.

Because the CCN score is based on a moderate number of gene features (i.e. 1979 gene pairs consisting of 1689 unique genes) relative to the total number of protein-coding genes in the genome, it is possible that a cancer model with a high CCN score might not have a high global similarity to a naturally occurring tumor. Therefore, we also calculated the gene regulatory network (GRN) status, a metric of the extent to which tumor type-specific gene regulatory network is established [54], for all models (Additional file 6: Fig. S5). We observed a high level of correlation between the two similarity metrics, which suggests that although CCN classifies on a selected set of genes, its scores are highly correlated with a more global assessment of transcriptional similarity.

We also sought to compare model modalities in terms of the diversity of subtypes that they represent (Additional file 6: Fig. S6). As a reference, we also included in this analysis the overall subtype incidence, as approximated by incidence in TCGA. Replicates in GEMMs and tumoroids were averaged into one classification profile. In models of UCEC, there is a notable difference in endometroid incidence, and the proportion of models classified as endometroid, with only PDX and tumoroids having any representatives (Additional file 6: Fig. S6). All of the CCL, GEMM, and tumoroid models of PAAD have an unknown subtype classification and no correct general classification. However, the majority of PDXs are subtyped as either a mixture of basal and classical, or classical alone. LUAD have proximal inflammation and proximal proliferation subtypes modeled by CCLs, PDXs, and tumoroids (Additional file 6: Fig. S6). Likewise, LUSC have classical and primitive subtypes modeled by CCLs, PDXs, and tumoroids, and whereas the secretory subtype is modeled by GEMMs exclusively and the basal subtype is modeled by PDXs exclusively (Additional file 6: Fig. S6). Taken together, these results demonstrate the need to carefully select different model systems to more suitably model certain cancer subtypes.

## Discussion

A major goal in the field of cancer biology is to develop models that mimic naturally occurring tumors with enough fidelity to enable therapeutic discoveries. However, methods to readily measure the extent to which cancer models resemble or diverge from native tumors are lacking. This is especially problematic now because there are many existing models from which to choose, and it has become easier to generate new models. Here, we present CancerCellNet (CCN), a computational tool that measures the similarity of cancer models to 22 naturally occurring tumor types and 36 subtypes. While the similarity of CCLs to patient tumors has already been explored in previous work, our tool introduces the capability to assess the transcriptional fidelity of PDXs, GEMMs, and tumoroids. Because CCN is platform- and species-agnostic, it represents a consistent platform to compare models across modalities including CCLs, PDXs, GEMMs, and tumoroids. Here, we applied CCN to 657 cancer cell lines, 415 patient-derived xenografts, 26 distinct genetically engineered mouse models, and 131 tumoroids. Several insights emerged from our computational analyses that have implications for the field of cancer biology.

First, PDXs have the greatest potential to achieve transcriptional fidelity with three out of five general tumor types for which data from all modalities was available, as indicated by the high scores of individual PDXs. Notably, PDXs are the only modality with samples classified as PAAD. At the same time, the median CCN scores of PDXs were lower than that of GEMMs and tumoroids in the other four tumor types. It is unclear what causes such a wide range of CCN scores within PDXs. We suspect that some PDXs might have undergone selective pressures in the host that distort the progression of genomic alterations away from what is observed in natural tumor [90]. Future work to understand this heterogeneity is important so as to yield consistently high fidelity PDXs, and to identify intrinsic and host-specific factors that so powerfully shape the PDX transcriptome.

Second, in general, GEMMs and tumoroids have higher median CCN scores than those of PDXs and CCLs. This is also consistent with that fact that GEMMs are typically derived by recapitulating well-defined driver mutations of natural tumors, and thus, this observation corroborates the importance of genetics in the etiology of cancer [91]. Moreover, in contrast to most PDXs, GEMMs are typically generated in immune replete hosts. Therefore, the higher overall fidelity of GEMMs may also be a result of the influence of a native immune system on GEMM tumors [92]. The high median CCN scores of tumoroids can be attributed to several factors including the increased mechanical stimuli and cell-cell interactions that come from 3D self-organizing cultures [93, 94].

Third, we have found that none of the samples that we evaluated here are transcriptionally adequate models of ESCA. This may be due to an inherent lability of the ESCA transcriptome that is often preceded by a metaplasia that has obscured determining its cell type(s) of origin [95]. Therefore, this tumor type requires further attention to derive new models.

Fourth, we found that in several tumor types, GEMMs tend to reflect mixtures of subtypes rather than conforming strongly to single subtypes. The reasons for this are not clear but it is possible that in the cases that we examined the histologically defined subtypes have a degree of plasticity that is exacerbated in the murine host environment.

Lastly, we recognize that many CCLs are not classified as their annotated labels. While we have suggested that the lack of immune component is not a major confounder for CCN, we suspect that the CCLs could undergo genetic divergence due to a high number of passages, chemotherapy before biopsy, culture condition, and genetic instability [96–99], which could all be factors that drive CCLs away from their labeled tumors. Furthermore, a recent study has proposed several contributors to cell line mislabeling such as inaccurate assignment based on unclear anatomical features or mismatch during sampling and adaption steps [16].

Currently, there are several limitations to our CCN tool and caveats to our analyses, which indicate areas for future work and improvement. First, CCN is based on transcriptomic data but other molecular readouts of tumor state, such as profiles of the proteome [100], epigenome [101], non-coding RNA-ome [101], and genome [91] would be equally, if not more important, to mimic in a model system. Therefore, it is possible that some models reflect tumor behavior well, and because this behavior is not well predicted by transcriptome alone, these models have lower CCN scores. To both measure the extent that such situations exist, and to correct for them, we plan in the future to incorporate other omic data into CCN so as to make more accurate and integrated model evaluation possible. As a first step in this direction, we plan to incorporate DNA methylation and genomic sequencing data as additional features for our random forest classifier as this data is becoming more readily available for both training and cancer models. We expect that this will allow us to both refine our tumor subtype categories and it will enable more accurate predictions of how models respond to perturbations such as drug treatment.

A second limitation is that in the cross-species analysis, CCN implicitly assumes that orthologs are functionally equivalent. The extent to which they are not functionally equivalent determines how confounded the CCN results will be. This possibility seems to be of limited consequence based on the high performance of the normal tissue cross-species classifier and based on the fact that GEMMs have the highest median CCN scores (in addition to tumoroids).

A third caveat to our analysis is that there were many fewer distinct GEMMs and tumoroids than CCLs and PDXs. As more transcriptional profiles for GEMMs and tumoroids emerge, this comparative analysis should be revisited to assess the generality of our results.

A fourth caveat is that although the gene pairs selected through CCN provide predictive capabilities in the random forest classifier, they do not necessarily represent marker genes for individual cancer types. Other methods that do not entail gene pair selection performed well in our benchmarking analysis, and thus, these approaches may yield more readily derivable sets of marker genes. We note that a definitive comparison of tumor model assessment tools awaits a more comprehensive and experimentally backed study.

Finally, the TCGA training data is made up of RNA-seq from bulk tumor samples, which necessarily includes non-tumor cells, whereas the CCLs are by definition cell lines of tumor origin. Therefore, CCLs theoretically could have artificially low CCN scores due to the presence of non-tumor cells in the training data. This problem appears to be limited as we found no correlation between tumor purity and CCN score in the CCLE samples. However, this problem is related to the question of intra-tumor heterogeneity. We demonstrated the feasibility of using CCN and single-cell RNA-seq data to refine the evaluation of cancer cell lines contingent upon availability of scRNA-seq training data. As more training single-cell RNA-seq data accrues, CCN would be able to not only evaluate models on per cell type basis, but also based on cellular composition.

## Conclusions

In summary, we have assessed the transcriptional similarities of four types of cancer models (CCLs, PDXs, GEMMs, and tumoroids) to naturally occurring human tumors. We have made the results of our analyses available online so that researchers can easily explore the performance of selected models or identify the best models for any of the 22 general tumor types and the 36 subtypes presented here. To ensure that CCN is widely available, we have developed a free web application, which performs CCN analysis on user-uploaded data and allows for direct comparison of their data to the cancer models evaluated here. We have also made the CCN code freely available under an Open Source license and as an easily installable R package, and we are actively supporting its further development. Included in the web application are instructions for training CCN and reproducing our analysis. The documentation describes how to analyze models and compare the results to the panel of models that we evaluated here, thereby allowing researchers to immediately compare their models to the broader field in a comprehensive and standard fashion.

## Supplementary Information

**Additional file 1: Table S1.** Specific parameters used for the training of all CCN classifiers.

**Additional file 2: Table S2.** Gene-pairs selected for final training of CCN general, subtype classifiers and single-cell classifier.

**Additional file 3: Table S3.** Decision thresholds and the corresponding average precision, recall and F1 for the general classifier and subtype classifiers.

**Additional file 4: Table S4.** Training parameters used to train 16 cross-species classifiers.

**Additional file 5: Table S5.** Accessions of tumor microarray data used in validation.

**Additional file 6: Supplementary Figures S1-S6**.

**Additional file 7: Table S6.** General classification profiles of CCLs.

**Additional file 8: Table S7.** Subtype classification profiles of CCLs.

**Additional file 9: Table S8.** LUSC CCLs subtype comparison between CCN, Yu et al, Salvadores et al.

**Additional file 10: Table S9.** General classification profiles of PDXs.

**Additional file 11: Table S10.** Subtype classification profiles of PDXs.

**Additional file 12: Table S11.** General classification profiles of GEMMs.

**Additional file 13: Table S12.** Subtype classification profiles of GEMMs.

**Additional file 14: Table S13.** General classification profiles of tumoroids.

**Additional file 15: Table S14.** Subtype classification profiles of tumoroids.

## Data Availability

Training Data: TCGA datasets [20] are available at https://www.cancer.gov/tcga. Normal (non-cancerous) tissue bulk RNA-seq datasets [45] are available at https://github.com/pcahan1/CellNet. RNA-seq Validation Data: ICGC datasets [56] used for validation are available at https://dcc.icgc.org/. Mouse normal tissue bulk RNA-seq datasets [45] used for cross-species validation are available at https://github.com/pcahan1/CellNet. Microarray tumor validation data: Microarray tumor datasets used for validation are available in the GEO database: GSE36771 [102], GSE21653 [103], GSE20685 [104], GSE50948 [105], GSE23177 [106], GSE26639 [107], GSE12276 [108], GSE31448 [103], GSE32646 [109], GSE65194 [110], GSE42568 [111], GSE26682 [112], GSE17536 [113], GSE41328 [114], GSE33114 [115], GSE26906 [116], GSE39582 [117], GSE62080 [118], GSE20916 [119], GSE18088 [120], GSE17537 [113], GSE23878 [121], GSE60697 [122], GSE37892 [123], GSE30540 [124], GSE50161 [125], GSE4290 [126], GSE60184 [127], GSE36245 [128], GSE53733 [129], GSE32374 [130], GSE34824 [131], GSE41137 [132], GSE53757 [133], GSE46699 [134], GSE36895 [135], GSE2109, GSE45436 [136], GSE9843 [137], GSE6222 [138], GSE19665 [139], GSE41804 [140], GSE10245 [141], GSE12667 [142], GSE37745 [143], GSE19188 [144], GSE40595 [145], GSE12172 [146], GSE20565 [147], GSE18520 [148], GSE10971 [149], GSE51373 [150], GSE14001 [151], GSE26193 [152], GSE55512 [153], GSE42404 [154], GSE16515 [155], GSE17891 [156], GSE15471 [157], GSE22780, GSE32688 [158], GSE17951 [159], GSE32448 [160], GSE7307, GSE32982 [161], GSE3325 [162], GSE26910 [163], GSE55945 [164], GSE7553 [165], GSE10282 [166], GSE19293 [166], GSE19234 [167], GSE35640 [168], GSE22968 [169], GSE34599, and GSE23376. Cell Lines Query Data: The CCLE cell line microarray and RNA-seq data are available at https://portals.broadinstitute.org/ccle/data and the GEO database GSE36139 [28]. NCCIT RNA-expression profiles are available on the GEO database: GSE63570 [29]. PDX Query Data: PDX query datasets are from the Novartis Institutes for BioMedical Research PDX Encyclopedia (NIBR PDXE) and were generously provided by Gao et al. [19] GEMM Query Data: GEMM query datasets are available on the GEO database: GSE114601 [30], GSE73541 [31], GSE65665 [32], GSE117552 [33], GSE76078 [34], GSE102598 [35], GSE118252 [36], GSE102345 [37], GSE10911 [38], and GSE109020 [38]. Tumoroid Query Data: Tumoroid query datasets from the NCI patient-derived models repository (PDMR) [39] are available at https://pdmr.cancer.gov/. The other tumoroid datasets are available in the GEO database: GSE84073 [40], GSE103990 [41], and GSE109982 [42]. Single Cell RNA-seq Data: Single-cell datasets used in this paper are available in the GEO database: GSE115978 [49] and GSE84465 [50].
