# Supplementary material for: Evaluating the transcriptional fidelity of cancer models
Source: Genome Med. 2021 Apr 29;13:73. doi: 10.1186/s13073-021-00888-w (PMC8086312; doi:10.1186/s13073-021-00888-w)
Supplement: Supplementary file 6 — Additional file 6: Supplementary Figures S1-S6. [file 13073_2021_888_MOESM6_ESM.pdf]

Supplementary Figures

Fig. S1 Optimization of CCN and comparison to alternative approaches.

Fig. S2 Assessment of CCN subtype classifiers.

Fig. S3 Further validation of CCN and CCLs classification results.

Fig. S4 Single-cell classification of SKCM and GBM cell lines.

Fig. S5 Correlation between cancer type specific gene regulatory network (GRN) status and general CCN scores.

Fig. S6 Proportions of cancer subtypes in different cancer models and TCGA tumor data across 11 general cancer types.

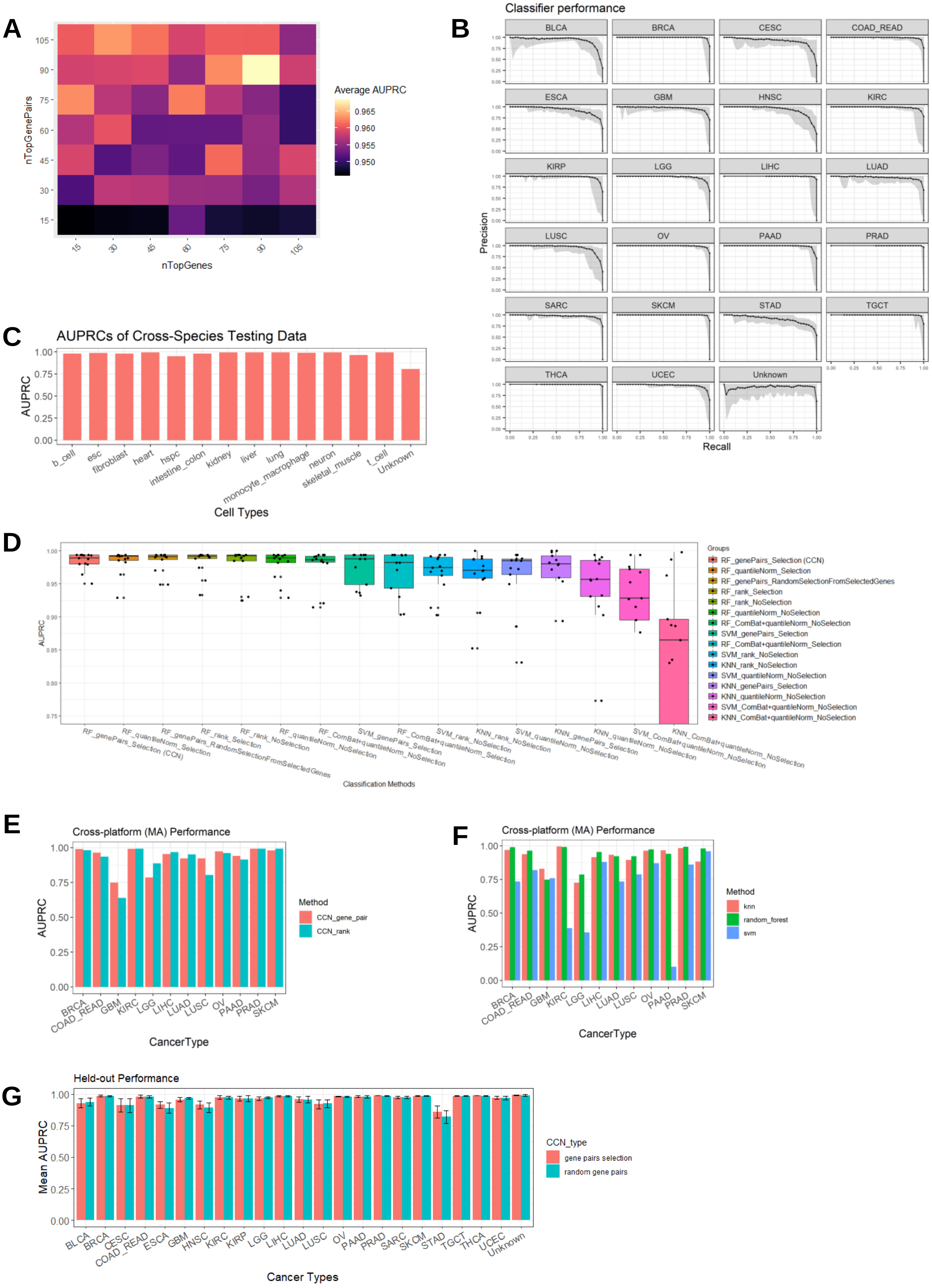

A
Average AUPRC
0.965
0.960
0.955
0.950
nTopGenePairs
105
90
75
60
45
30
15
nTopGenes
15
30
45
60
75
90
105
B
Classifier performance
BLCA
BRCA
CESC
COAD_READ
ESCA
GBM
HNSC
KIRC
KIRP
LGG
LIHC
LUAD
LUSC
OV
PAAD
PRAD
SARC
SKCM
STAD
TGCT
THCA
UCEC
Unknown
Precision
Recall
0.00
0.25
0.50
0.75
1.00
C
AUPRCs of Cross-Species Testing Data
AUPRC
1.00
0.75
0.50
0.25
0.00
b_cell
esc
fibroblast
heart
hspc
intestine_colon
kidney
liver
lung
monocyte_macrophage
neuron
skeletal_muscle
t_cell
Unknown
Cell Types
D
AUPRC
1.00
0.95
0.90
0.85
0.80
0.75
Groups
RF_genePairs_Selection (CCN)
RF_quantileNorm_Selection
RF_genePairs_RandomSelectionFromSelectedGenes
RF_rank_Selection
RF_rank_NoSelection
RF_quantileNorm_NoSelection
RF_ComBat+quantileNorm_NoSelection
SVM_genePairs_Selection
RF_ComBat+quantileNorm_Selection
SVM_rank_NoSelection
KNN_rank_NoSelection
SVM_quantileNorm_NoSelection
KNN_genePairs_Selection
KNN_quantileNorm_NoSelection
SVM_ComBat+quantileNorm_NoSelection
KNN_ComBat+quantileNorm_NoSelection
Classification Methods
E
Cross-platform (MA) Performance
AUPRC
1.00
0.75
0.50
0.25
0.00
Method
CCN_gene_pair
CCN_rank
BRCA
COAD_READ
GBM
KIRC
LGG
LIHC
LUAD
LUSC
OV
PAAD
PRAD
SKCM
CancerType
F
Cross-platform (MA) Performance
AUPRC
1.00
0.75
0.50
0.25
0.00
Method
knn
random_forest
svm
BRCA
COAD_READ
GBM
KIRC
LGG
LIHC
LUAD
LUSC
OV
PAAD
PRAD
SKCM
CancerType
G
Held-out Performance
Mean AUPRC
1.00
0.75
0.50
0.25
0.00
CCN_type
gene pairs selection
random gene pairs
BLCA
BRCA
CESC
COAD_READ
ESCA
GBM
HNSC
KIRC
KIRP
LGG
LIHC
LUAD
LUSC
OV
PAAD
PRAD
SARC
SKCM
STAD
TGCT
THCA
UCEC
Unknown
Cancer Types

Fig. S1 Optimization of CCN and comparison to alternative approaches. **(A)** Mean AUPRC of repeated grid-search cross-validation for each parameter grid. **(B)** Mean and range of CCN classifier's PR curves from 50 cross validations based on the optimal feature selection parameters nTopGenes = *90* and nTopGenePairs = *90.* **(C)** AUPRCs of CCN human tissue classifier when applied to mouse tissue data. **(D)** Benchmarking of CCN with 15 other methods using different combinations of classification algorithm, data transformation and feature selections. **(E)** Cross-platform (microarray) performance comparison of CCN classifiers trained using gene pairs and gene ranks. **(F)** Cross-platform (microarray) performance comparison between gene pair KNN, gene pair Random Forest and gene pair SVM. **(G)** Performance of CCN classifiers trained with selected gene pairs and random gene pairs across 20 cross-validations.

A

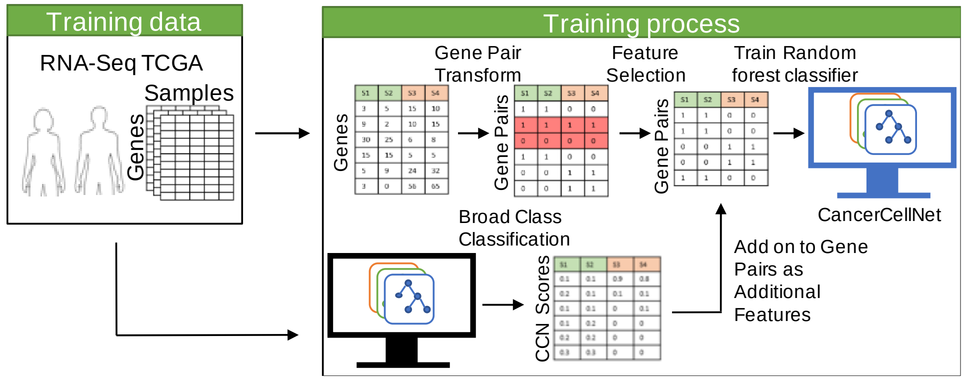

B

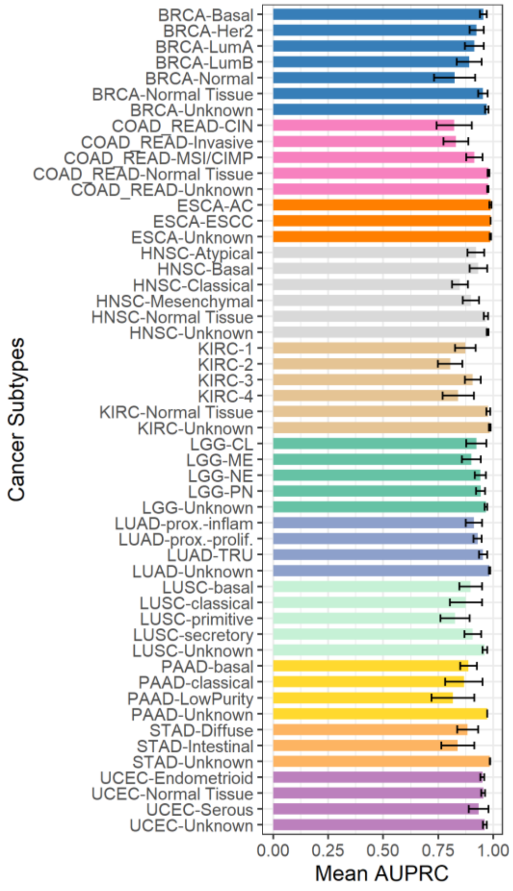

Fig. S2 Assessment of CCN subtype classifiers. **(A)** The schematic of training a subtype classifier in CCN. CCN uses patient tumor expression profiles from cancer of interest as training data. CCN performs gene-pair transformation and selects the most discriminative gene pairs among the cancer subtypes from training data as features. CCN then applies the general classification on training data and uses the general classification profiles as features in addition to gene pairs for training a Random Forest classifier. The weight of the general classification profiles as features can be tuned to improve AUPRCs. **(B)** The mean and standard deviation of AUPRCs for 11 subtype classifiers based on 20 cross-validations.

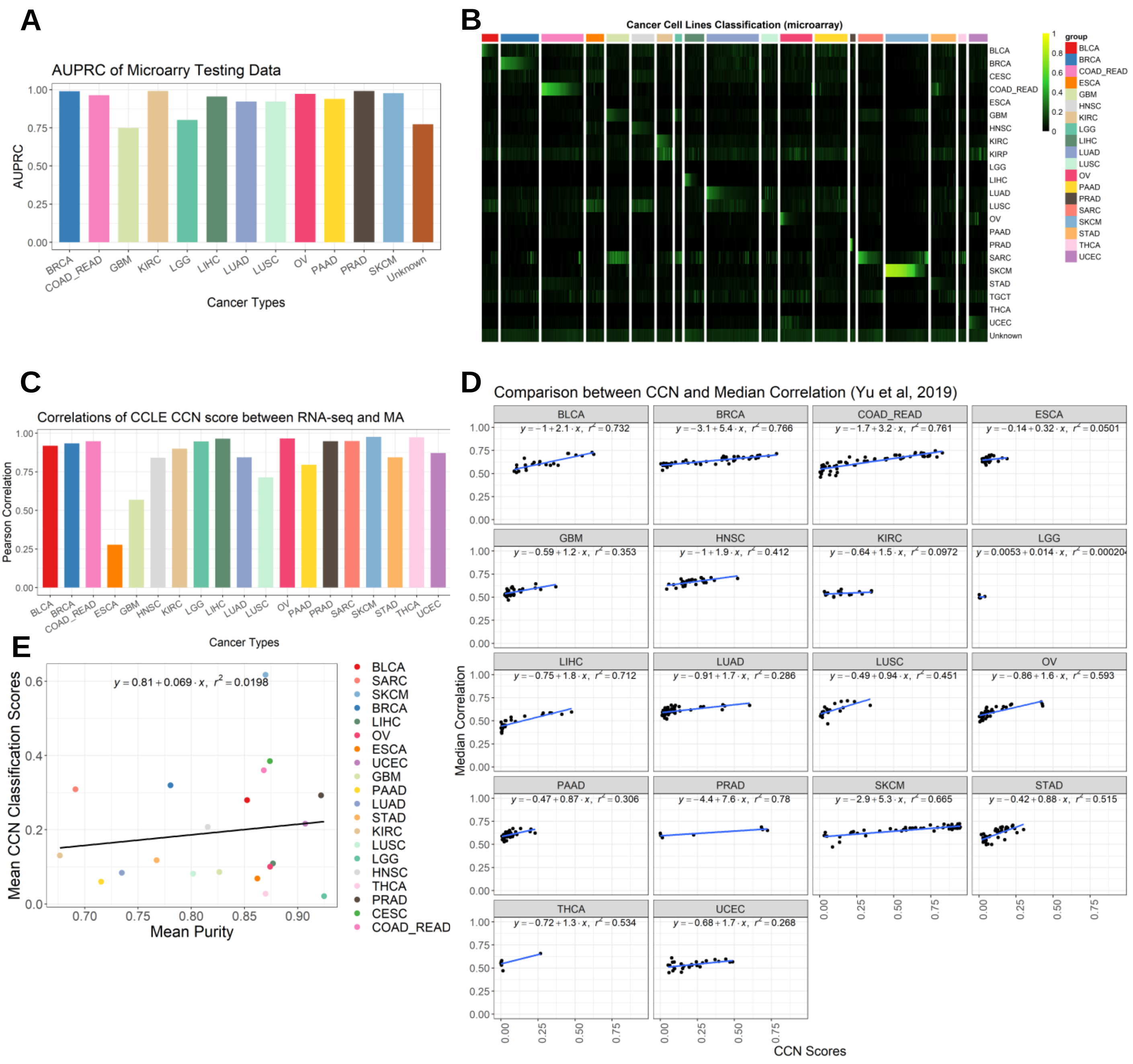

Fig. S3 Further validation of CCN and CCLs classification results. To validate the cross-platform classification performance of CCN, a new classifier specifically used to classify microarray data was trained using RNA-seq data from TCGA as training data and intersecting genes between RNA-seq data and microarray data. **(A)** AUPRCs of CCN classifier when applied to tumor profiles assayed on microarray. **(B)** Classification heatmap of CCLs using microarray expression data. **(C)** Pearson correlation between CCN scores of CCLE lines generated from RNA-seq data and microarray data. **(D)** Comparison between CCLs' CCN scores and the median correlation metric from Yu et al. **(E)** Comparison of mean tumor purity of training data and mean CCN scores of CCLs for each cancer category.

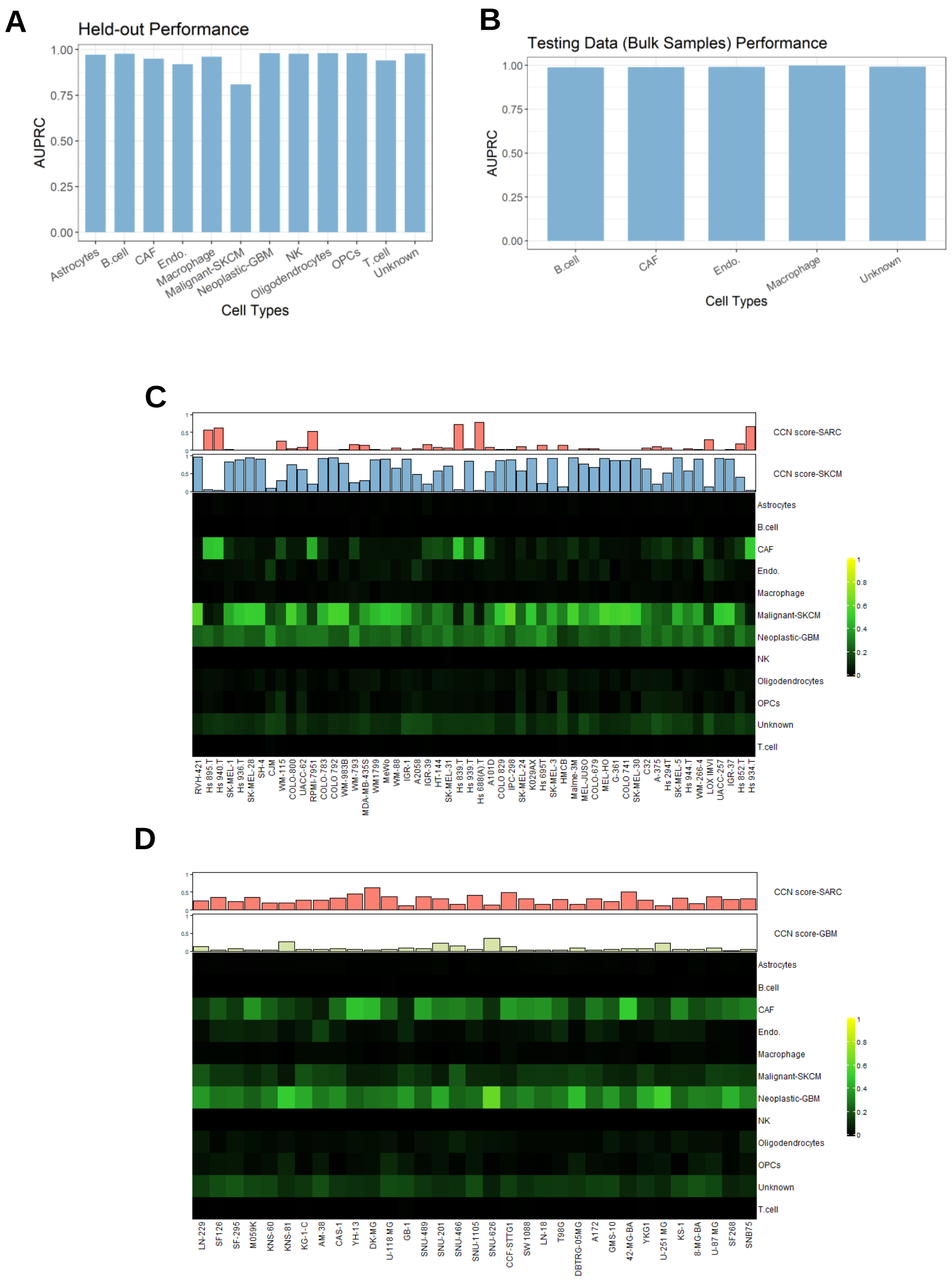

Fig. S4 Single-cell classification of SKCM and GBM cell lines. **(A)** AUPRCs of the single-cell classifier when applied to scRNA-seq held-out data. **(B)** AUPRCs of the scRNA-seq classifier when applied to purified bulk RNA samples. **(C)** Single-cell classification of SKCM CCLs. Red bar plot (top) represents general CCN scores in SARC and blue bar plot (bottom) represents general CCN scores in SKCM. **(D)** Single-cell classification of GBM CCLs. Red bar plot (top) represents general CCN scores in SARC and yellow bar plot (bottom) represents general CCN scores in GBM.

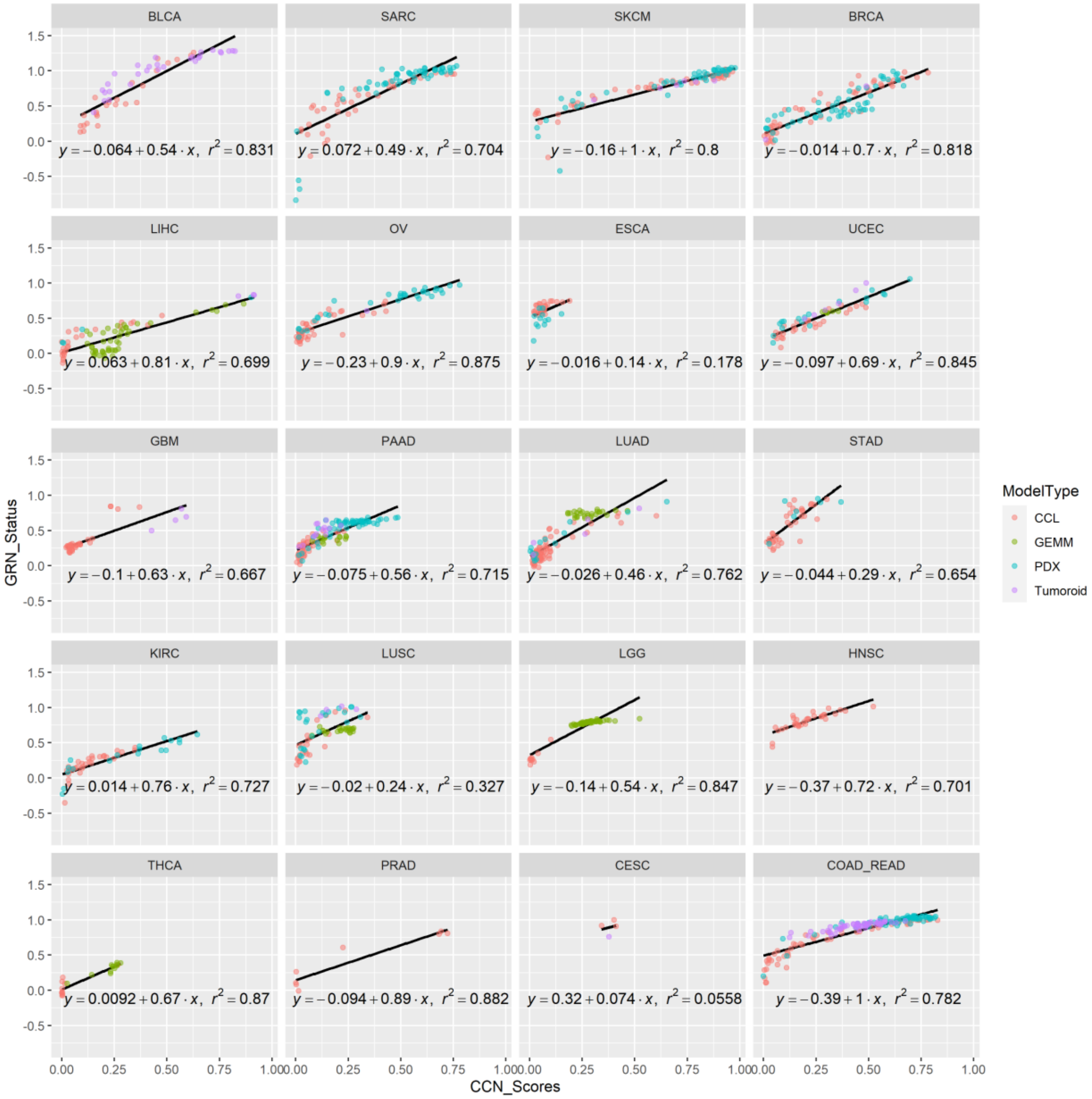

Fig. S5 Correlation between cancer type specific gene regulatory network (GRN) status and general CCN scores.

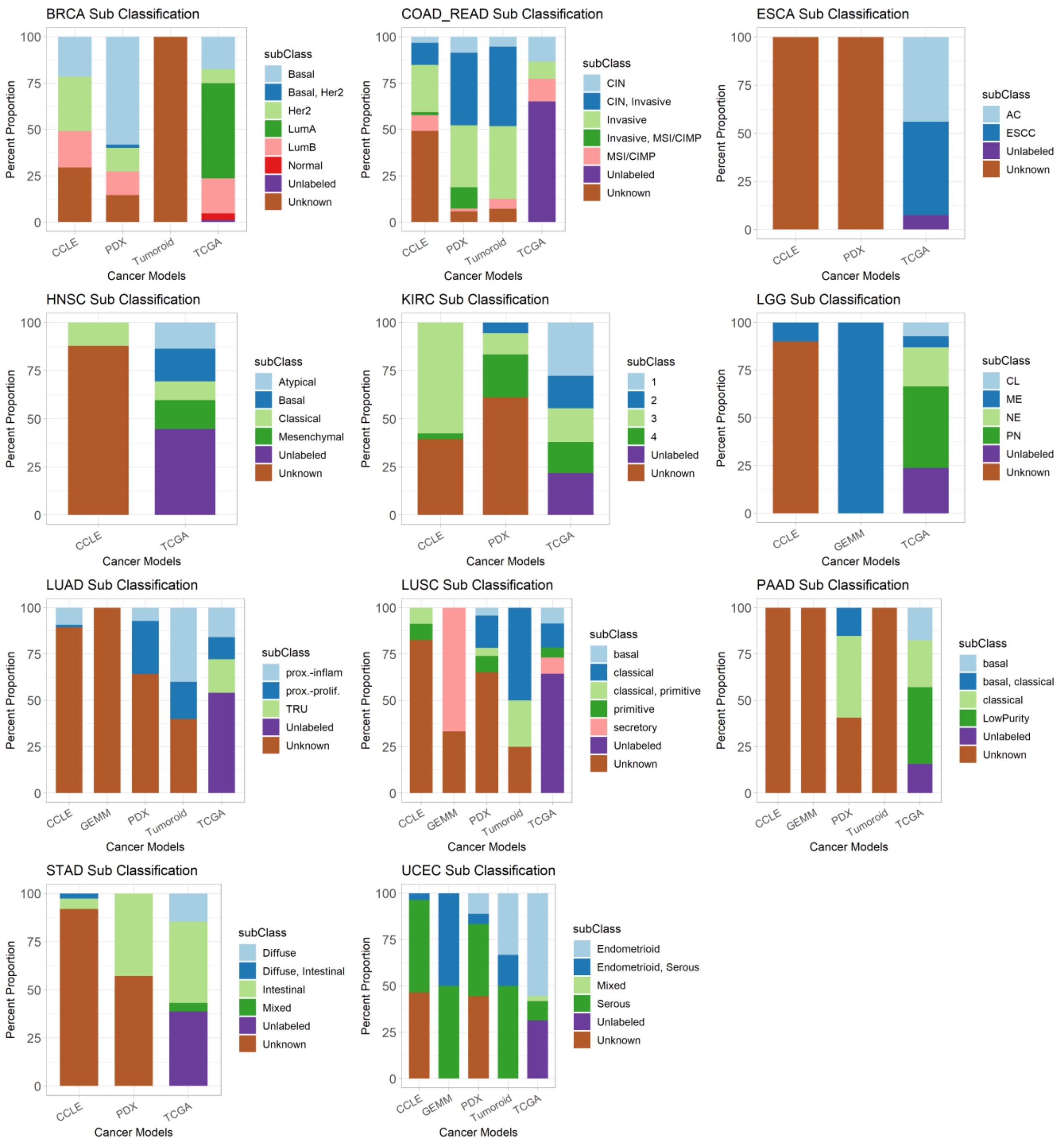

Fig. S6 Proportions of cancer subtypes in different cancer models and TCGA tumor data across 11 general cancer types.
